# Structural conservation of Lassa virus glycoproteins and recognition by neutralizing antibodies

**DOI:** 10.1016/j.celrep.2023.112524

**Published:** 2023-05-18

**Authors:** Hailee R. Perrett, Philip J.M. Brouwer, Jonathan Hurtado, Maddy L. Newby, Lin Liu, Helena Müller-Kräuter, Sarah Müller Aguirre, Judith A. Burger, Joey H. Bouhuijs, Grace Gibson, Terrence Messmer, John S. Schieffelin, Aleksandar Antanasijevic, Geert-Jan Boons, Thomas Strecker, Max Crispin, Rogier W. Sanders, Bryan Briney, Andrew B. Ward

**Affiliations:** 1 Department of Integrative, Structural and Computational Biology, The Scripps Research Institute, La Jolla, CA 92037, USA; 2 Department of Immunology and Microbiology, Scripps Research, La Jolla, CA 92037, USA; 3 Center for Viral Systems Biology, Scripps Research, La Jolla, CA 92037, USA; 4 School of Biological Sciences, University of Southampton, Southampton SO17 1BJ, UK; 5 Complex Carbohydrate Research Center, University of Georgia, Athens, GA 30602, USA; 6 Institute of Virology, Philipps University Marburg, 35043 Marburg, Germany; 7 Department of Medical Microbiology and Infection Prevention, Amsterdam University Medical Centers. Location AMC, University of Amsterdam, Amsterdam Infection & Immunity Institute, Amsterdam 1105 AZ, the Netherlands; 8 Department of Pediatrics, Tulane University School of Medicine, New Orleans, LA 70112, USA; 9 Department of Chemical Biology and Drug Discovery, Utrecht University, Utrecht 3584 CG, the Netherlands; 10 Department of Microbiology and Immunology, Weill Medical College of Cornell University, New York, NY 10021, USA

**Keywords:** Lassa mammarenavirus, Lassa fever, arenavirus, structure-based vaccine design, cryo-EM, neutralizing antibody, prefusion glycoprotein

## Abstract

Lassa fever is an acute hemorrhagic fever caused by the zoonotic Lassa virus (LASV). The LASV glycoprotein complex (GPC) mediates viral entry and is the sole target for neutralizing antibodies. Immunogen design is complicated by the metastable nature of recombinant GPCs and the antigenic differences among phylogenetically distinct LASV lineages. Despite the sequence diversity of the GPC, structures of most lineages are lacking. We present the development and characterization of prefusion-stabilized, trimeric GPCs of LASV lineages II, V, and VII, revealing structural conservation despite sequence diversity. High-resolution structures and biophysical characterization of the GPC in complex with GP1-A-specific antibodies suggest their neutralization mechanisms. Finally, we present the isolation and characterization of a trimer-preferring neutralizing antibody belonging to the GPC-B competition group with an epitope that spans adjacent protomers and includes the fusion peptide. Our work provides molecular detail information on LASV antigenic diversity and will guide efforts to design pan-LASV vaccines.

## Highlights

• Stabilization of diverse Lassa virus glycoproteins enables their structural characterization
• mAb 12.1F, belonging to the GP1-A cluster, inhibits matriglycan and LAMP-1 binding
• GP1-A mAbs show glycan dependence with 19.7E demonstrating lineage-dependent binding
• A trimer-preferring Ab S370.7 targets the GPC-B epitope

## Graphical abstract

**Figure:**
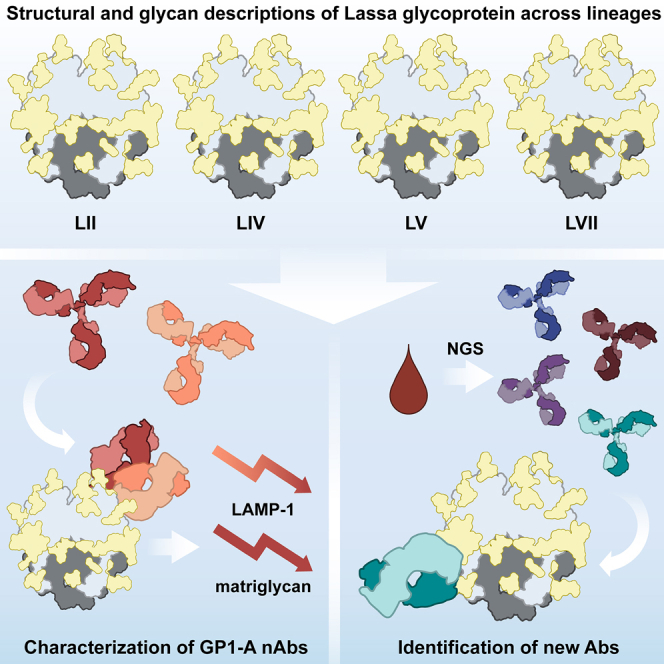

## Teaser

Perrett et al. structurally characterize Lassa glycoproteins from four lineages (II, IV, V, and VII) and determine the molecular interactions critical for GP1-A NAb engagement. They then use these stable glycoproteins to isolate an antibody from convalescent patient serum.

## Introduction

The ongoing SARS-CoV-2 pandemic emphasizes the importance of pandemic preparedness for zoonotic pathogens, which—through climate and anthropogenic variables that increase the landscape suitability for zoonotic transmission—cause approximately 75% of infectious disease in humans.^1^^,^^2^ Since its identification in 1969, the Old World mammarenavirus Lassa (LASV [Lassa virus]) has caused endemic Lassa fever disease in West Africa. While most cases appear to be asymptomatic,^3^ an acute hemorrhagic fever can develop, leading to high case fatality ratios often exceeding 25% among patients showing clinical symptoms.^4^^,^^5^^,^^6^ LASV is most often transmitted to humans from spillover events with its near-ubiquitous reservoir host *Mastomys natalensis*, which is otherwise known as the natal multimammate rat. Transmission more rarely occurs via nosocomial infection^7^ and sexual transmission post-recovery.^8^^,^^9^ Because of its substantial genomic variability, LASV is subdivided into seven distinct genetic lineages (I–VII).^10^^,^^11^^,^^12^ This variability increases the difficulty of developing robust diagnostics, likely resulting in an underrepresentation of LASV’s disease toll.^13^^,^^14^^,^^15^ There are no efficacious treatments or vaccines for this disease except the controversial off-label use of ribavirin and supportive care.^16^ Owing to this, the World Health Organization and the Coalition for Epidemic Preparedness Innovations recognize the need for increased LASV research and development efforts given its pandemic potential^17^ and have supported early-stage vaccine development and corresponding clinical trials.^18^

The glycoprotein complex (GPC) is the only viral protein on the surface of LASV and presents the sole target for neutralizing antibodies (NAbs).^19^^,^^20^ The GPC is expressed as a single polypeptide precursor before being proteolytically processed by signal peptidase (SPase)^21^ followed by site-1 protease (S1P).^22^ In its mature, native form, GPC is a trimer of heterotrimers comprised of the receptor-engaging subunit GP1; the transmembrane-spanning subunit GP2, which contains the GPC’s fusion peptide at its N terminus^23^; and the stable signal peptide (SSP), which remains non-covalently complexed near the membrane-proximal region of GP2 post-cleavage.^24^ Approximately 25% of the GPC molecular weight is attributable to the highly conserved 11 or 12—lineage-depending—potential N-linked glycosylation sites (PNGSs) per monomer.^20^^,^^25^ As a result, the GPC carries a dense glycan shield, which contributes to LASV’s evasion of neutralizing humoral immune responses.^26^ Similar to the HIV-1 Env, the LASV GPC features a cluster of oligomannose-type glycans^20^ that function as an attachment factor and enable LASV’s infection of immune cells via the C-type lectin DC-SIGN (dendritic cell-specific intercellular adhesion molecule-3-grabbing nonintegrin).^27^ Shedding of GP1 during acute disease in humans has been observed and is thought to act as an immune decoy given the conformational variability between shed GP1 and GP1 presented as part of the GPC.^28^^,^^29^^,^^30^ LASV exploits several host cell receptors to infect human cells.^31^^,^^32^^,^^33^ Primary host cell attachment is mediated by matriglycan moieties on α-dystroglycan, which interact with residues on the GPC trimer apex.^24^^,^^34^^,^^35^^,^^36^ Upon macropinocytosis and trafficking of LASV through the endosomal compartments,^37^ GPC undergoes a pH-dependent switch, allowing binding to endosomal receptor lysosomal-associated membrane protein 1 (LAMP-1).^38^^,^^39^ Putative residues for LAMP-1 binding involve the histidine triad and supporting GP1 residues.^40^^,^^41^

The largest anti-LASV Ab isolation study to date, which yielded 113 cloned human monoclonal Abs (mAbs) from memory B cells of LASV survivors, defines the canonical Ab competition groups: GP1-A, GPC-A, GPC-B, and GPC-C.^19^ X-ray crystallography studies with GPC-B-specific mAbs revealed that the GPC-B epitope extends across two protomers at the base of the GPC trimer, making contacts with the N-terminal loop, the T-loop, HR1 and HR2 helices, and the fusion peptide.^30^^,^^42^ Cryo-electron microscopy (cryo-EM) structures have shown that the GPC-A epitope extends across the GP1 and GP2 subunits and is situated among the N79, N89, N99, N224, and N365 glycans.^43^ Both Ab competition groups have been shown to lower the fusogenicity of the GPC and limit binding to LAMP-1. More recent work describes the structural characterization of the GPC in complex with GP1-A-specific NAb 12.1F alongside mAbs 8.9F and 37.2D, which target the GPC-C and GPC-B epitopes, respectively.^44^ Together, these three Abs are used in the Arevirumab-3 cocktail, which shows remarkable efficacy in protecting cynomolgus macaques at advanced stages of disease.^45^

Previous structural work of a ligand-free, native-like GPC has been made difficult by the instability of the trimeric ectodomain^30^ and inefficient cleavage when introducing stabilization mechanisms.^35^^,^^46^^,^^47^^,^^48^ Published structural information of the GPC in its prefusion conformation is mostly limited to the GPC from the lineage IV Josiah strain in complex with Abs,^24^^,^^30^^,^^42^^,^^43^ although a recent study describes the GPC from lineage I.^49^ Our recent work demonstrates that fusing the GPC to the I53-50A component (GPC-I53-50A) of the computationally designed I53-50 nanoparticle^50^ stabilized the trimeric conformation of the GPC.^51^ In line with the generation of I53-50 nanoparticles presenting glycoproteins of HIV-1, SARS-CoV-2, and respiratory syncytial virus (RSV), GPC-I53-50 nanoparticles assembled efficiently upon mixing of GPC-I53-50A and the pentameric subunit I53-50B.^51^^,^^52^^,^^53^ Display of the GPC on I53-50 nanoparticles has demonstrated success in eliciting NAb responses and *in vivo* protection, yet the full nanoparticle system complicates structural analysis.^51^

Here, we utilize the I53-50A subunit as a scaffold to generate and characterize GPC trimers of LASV genotypes beyond the prototypical lineage IV strain Josiah. We focus on LASV lineages of public health concern including lineage II, one of the most common lineages that circulates widely in southern Nigeria; lineage V, which circulates in Mali and has decreased pathogenicity compared with lineage IV; and lineage VII, a newly described lineage isolated from a nosocomial infection in Togo with comparable pathogenicity to lineage IV.^11^^,^^54^^,^^55^ Establishing a single-particle cryo-EM GPC pipeline allowed us to generate unliganded high-resolution structures of these GPC trimers, revealing structural commonalities and subtle differences between these geographically distinct LASV lineages. In addition, we present the structures of GPC-I53-50A in complex with NAbs 19.7E and 12.1F, adding molecular details to GP1-A-targeting mAbs’ epitopes and context to their different neutralization phenotypes. Finally, we describe the isolation and structural characterization of a trimer-preferring mAb from a survivor of Sierra Leonean Lassa fever, providing additional molecular information for the GPC-B epitope cluster. This work not only expands our structural knowledge of the different LASV lineages and their NAb epitopes but also enables investigation of lineage antigenicity at the molecular level—critical steps toward the development of a pan-LASV vaccine.

## Results

### Engineering stable prefusion LASV GPC trimers of different lineages

**Figure 1:**
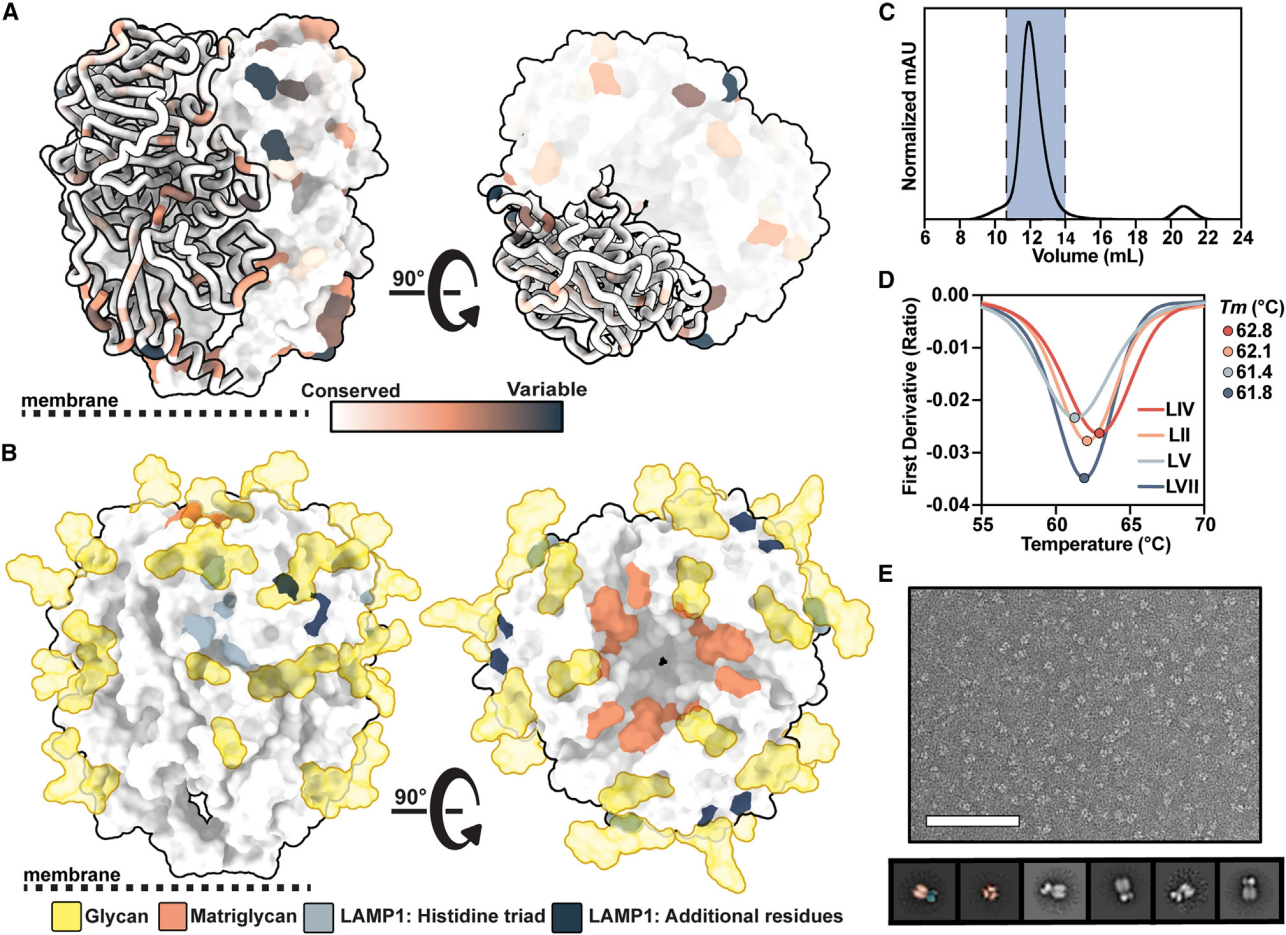
Biophysical characterization of LASV GPCs derived from diverse lineages and scaffolded on I53-50A (A) LASV GPC sequence conservation mapped on ribbon and surface representation of the LIV GPC (PDB: 8EJD). Residues with increasing sequence variability are depicted in orange and dark blue. (B) Glycans modeled from experimental density (gold; PDB: 8EJD), residues involved in matriglycan binding (orange),^24^ and residues suspected in LAMP-1 binding (histidine triad in gray, additional residues in navy blue^40^^,^^41^) mapped on the surface representation of the LIV GPC. (C) Representative size-exclusion chromatogram (SEC) of GPC-I53-50A. Fractions containing GPC-I53-50A trimer are shown in blue. (D) Thermostability of GPC-I53-50As assessed by the inflection point of the ratio of signal at 350 and 330 nm, as measured by nanoDSF. Circles mark the midpoint of thermal denaturation, or melting temperature (*T*_m_), of each protein, with values listed on the right of the graph. Each melting curve is a representative of triplicate curves with *T*_m_ within ±0.1°C. (E) Raw negative stain EM image (top) of the SEC-purified LIV GPC-I53-50A. Scale bar represents 200 nm. 2D class averages (bottom) of the GPC-I53-50A are shown with the left two classes pseudocolored to represent the GPC (orange) and I53-50A scaffold (blue).

As LASV has known antigenic differences that may affect humoral cross-reactivity,^10^^,^^12^ we first assessed the sequence conservation of LASV’s GPC across >350 databank sequences derived from human and rodent field isolates.^56^^,^^57^ While the GPCs have highly conserved sequences in the receptor-binding sites and PNGSs, there is notable variability (Figures 1A and 1B). To study these antigenic distinctions at a molecular level, we expanded our repertoire of recombinant trimeric GPCs. Building on our previous success using the I53-50A scaffold to stabilize the Josiah strain GPC ectodomain, we explored whether the same strategy could stabilize additional GPC trimers of diverse LASV lineages.^51^ To ensure stabilization of the prefusion state, we introduced the GPCysR4 mutations.^30^ These mutations comprise the introduction of a disulfide bond between GP1 and GP2, a proline in the HR1 helix, and the replacement of the native LASV GPC S1P cleavage site^22^^,^^58^ with a furin cleavage site. The resulting soluble constructs feature sequences of circulating lineages II (LII; strain NIG08-A41), V (LV; strain Soromba-R), and VII (LVII; strain Togo/2016/7082). GPC-I53-50As were expressed using codon-optimized plasmids in HEK 293F cells. Our combined analysis using size-exclusion chromatography, nano differential scanning fluorimetry (nanoDSF), and negative stain EM reveals that the expressed constructs form homogeneous, prefusion trimers with comparable thermostability (Figures 1C–1E, S1A, and S1B).

### GPCs from diverse LASV lineages have similar glycan shields

**Figure 2:**
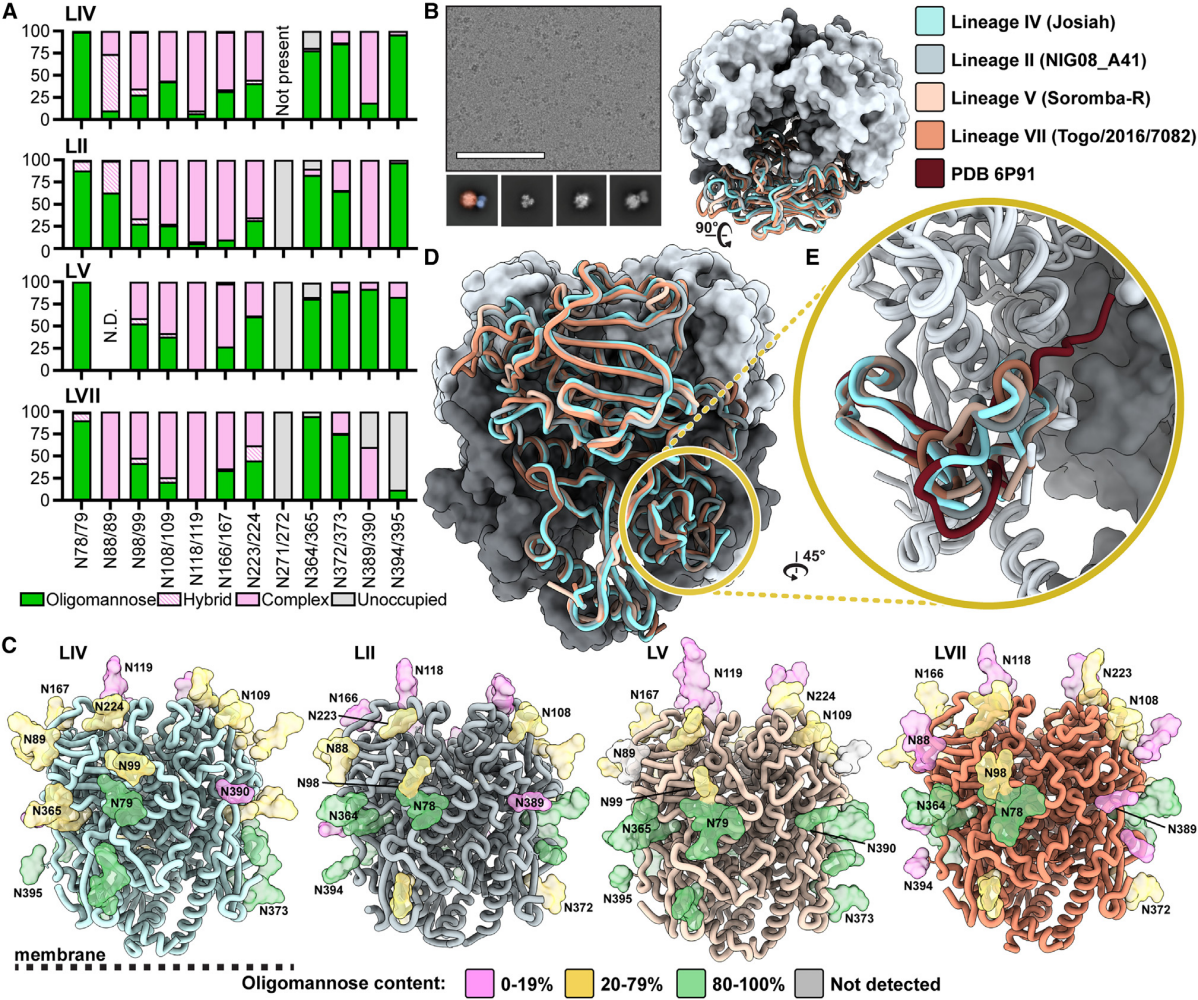
Site-specific glycosylation and structural analysis of LASV GPCs from different lineages (A) Relative quantification of distinct glycan types of GPC-I53-50As determined by LC-MS describe the relative glycan processing state at a particular PNGS. Oligomannose-type glycans are shown in green, hybrid in dashed pink, and complex glycans in pink. Unoccupied sites are shown in gray. (B) Representative micrograph of ligand-free GPC-I53-50A. Sample 2D classes are shown below, with the leftmost class pseudocolored to indicate the GPC (orange) and I53-50A trimerization scaffold (blue). Scale bar represents 100 nm. (C) Refined atomic models of ligand-free LASV GPC structures of LIV (strain Josiah), LII (strain NIG08-A41), LV (strain Soromba-R), and LVII (strain Togo/2016/7082). Glycans are shown as colored surfaces according to their oligomannose content. Though MS data show N394 on the LVII GPC as primarily unoccupied, it is colored according to its glycan identity when present since the PNGS site was observed in the EM data. Access codes are as follows: LIV, PDB: 8EJD, EMDB: EMD-28178; LII, PDB: 8EJE, EMDB: EMD-28179; LV, PDB: 8EJF, EMDB: EMD-28180; and LVII, PDB: 8EJG, EMDB: EMD-28181. (D) Comparison of models in (C). (E) Comparison of fusion peptides (LIV and LV residues 260–299; LII and LVII residues 259–298) of models in (C) with PDB: 6P91,^42^ which features the LIV GPC in complex with 18.5C Fab.

LASV GPC has a highly dense glycan shield^20^ that preferentially envelops GP1 over GP2, resulting in just under 50% of GP1's surface area being shielded by glycans.^59^ The prototypical LIV strain Josiah GPC exhibits 11 PNGSs on its GPC ectodomain,^20^^,^^25^ which are thought to contribute to host immune evasion.^60^ LII, LV, and LVII each have an additional PNGS at residue N271 or N272, though this site is uniformly unoccupied (Figure 2A). Glycan analysis via liquid chromatography-mass spectrometry (LC-MS) reveals a large range of glycan processing states with a notable abundance of oligomannose-type glycans near the N- and C-terminal regions of the GPC. Complex-type glycans were presented at a higher rate on centrally located PNGSs. Glycan microheterogeneity is pronounced at sites N98/99, N166/167, and N223/224, with each site presenting a mix of oligomannose-, hybrid-, and complex-type glycoforms. This microheterogeneity is largely conserved between lineages (Figure 2A). The N118/119 site displays almost exclusively complex-type glycans, all of which are fucosylated (Figure S2A). The GPC’s glycan shield features an unusual mannose patch similar to HIV-1 Env,^61^^,^^62^^,^^63^ which is likely caused by steric constraints from neighboring glycan moieties. This restricts access of these PNGSs to glycan processing enzymes in the endoplasmic reticulum and Golgi apparatus.^20^ Previous analysis in a virus-like particle system denotes the mannose patch of the LASV GPC as PNGSs N79, N89, N99, N365, and N373,^20^ yet the lineages presented in Figure 2A show a large proportion of complex-type glycans presented at N89 and N99. This distinction may be attributable to the different expression systems used to generate the GPCvirus-like particles (Madin-Darby canine kidney II cells) or recombinant GPC-I53-50As (HEK 293F cells). Alternatively, the differences may be explained by variation in the oligomerization and/or cleavage efficiencies of the GPCs expressed on the membrane or as recombinant proteins.

### GPCs from diverse lineages demonstrate similar structural features with a distinct fusion peptide conformation

We next assessed whether the LASV lineages present GPCs with distinct structural features. Using single-particle cryo-EM, we optimized the conditions for freezing LIV, LII, LV, and LVII GPC-I53-50A trimers (Figure 2B). Because the GPC is highly glycosylated and has less accentuated features compared with other viral fusion glycoproteins, we found it was difficult to (1) overcome the strong orientation bias of the GPC particle in vitreous ice and (2) align the GPC during data processing when we masked out densities outside of the GPC. Orientation bias likely caused by the interactions of the apex glycans with the air-water interface was relieved by adding a fluorinated detergent to the sample prior to freezing (Figure S3A). To alleviate poor alignment, we began processing the data using the I53-50A scaffold as a fiducial marker, which facilitated better orientation of the GPC. Combined, these approaches enabled us to determine the structures of GPC in a reproducible manner and yielded structures of LIV, LII, LV, and LVII GPC trimers at resolutions of 3.8, 3.7, 3.7, and 3.1 Å, respectively (Figures 2C and S3–S5; Table S1).

The GPC-I53-50A constructs recapitulate the known GPC structural features and domain organization.^30^ GP1 features N-terminal β-strands, the exterior β-sheet surface, and the interior helix-loop domain. The GP2 subunit demonstrates the canonical HR1a–d helices, T-loop, and HR2 helix. Our GPC-I53-50A shows high similarity, as measured by the root-mean-square deviation (RMSD) of a GPC protomer, to those previously described (0.80 Å among 300 pruned atom pairs, 2.4 Å overall fit across 341 sequence-aligned pairs with PDB: 5VK2^30^ and 1.1 Å among 254 pruned atom pairs, 2.9 Å across 341 sequence-aligned pairs with PDB: 7PVD^24^; Figure S6A). Similarly, when comparing the GPCs of diverse lineages, we observe high structural homology (Figure 2D). Using the prototypical LIV GPC as reference, we note RMSDs for pruned and all sequence-aligned pair as follows: 0.78 and 1.9 (LII), 0.85 and 2.0 (LV), and 0.73 and 2.4 Å (LVII).

The main differences among the GPC lineages are found in flexible loops, most notably the loop (residues 166–181) extending from the β7 sheet prior to the α3 helix (Figure S2B). This observed heterogeneity is derived from areas in the EM map of poorer local resolution (Figure S5), insinuating greater flexibility of the residues in these regions. Consequently, these differences may not represent physiologically important conformational epitopes for LII, LV, and LVII GPCs.

When comparing our structures with the Ab-bound structures reported previously,^42^ we observe a substantial difference in fusion peptide conformation (Figure 2E). In the ligand-free structures of GPC-I53-50As, the fusion peptide appears to flexibly occupy the space enclosed by the HR1a helix of the same protomer and the HR1d and HR2 loop of its adjacent protomer. In contrast, previously described structures of GPC bound to 18.5C, 37.7H, and 25.6A of the GPC-B competition group,^30^^,^^42^ 25.10C of the GPC-A competition group,^43^ and full-length GPC bound to matriglycan show that the fusion peptide occupies the same approximate area, yet extends inward and reaches toward the apex of the trimer near the GP1 C-terminal domain (Figures 2E, S6A, and S6B). This conformational difference increases the buried surface area of residues 260–276 of the fusion peptide from 598 to 621 Å^2^ upon 18.5C binding, for example, and lowers the solvent accessibility of the fusion peptide. Both the Ab-bound and unbound structures show the fusion peptides adopting a near-identical conformation starting at the fusion loop (residues 277–299).

### GP1-A-specific mAbs 12.1F and 19.7E neutralize by blocking receptor binding

While GPC-A- and GPC-B-specific Ab interactions with GPC have been studied in detail,^30^^,^^42^^,^^43^ molecular details of Abs targeting the GP1-A epitope have, until recently, remained elusive. Although 12.1F and 19.7E are both members of the described GP1-A competition cluster,^19^ these mAbs have distinct genetic features. Whereas the heavy chain (HC) and light chain (LC) of 12.1F are derived from IGHV4-34^∗^01 and IGKV3-11^∗^01, respectively, the germline HC and LC of 19.7E are IGHV3-74^∗^02 and IGKV1-5^∗^01. The VH genes of 12.1F HC and LC are 8.8% and 7.6% somatically hypermutated, respectively, based on the sequences publicly available (patent WIPO: WO2018106712A1).

**Figure 3:**
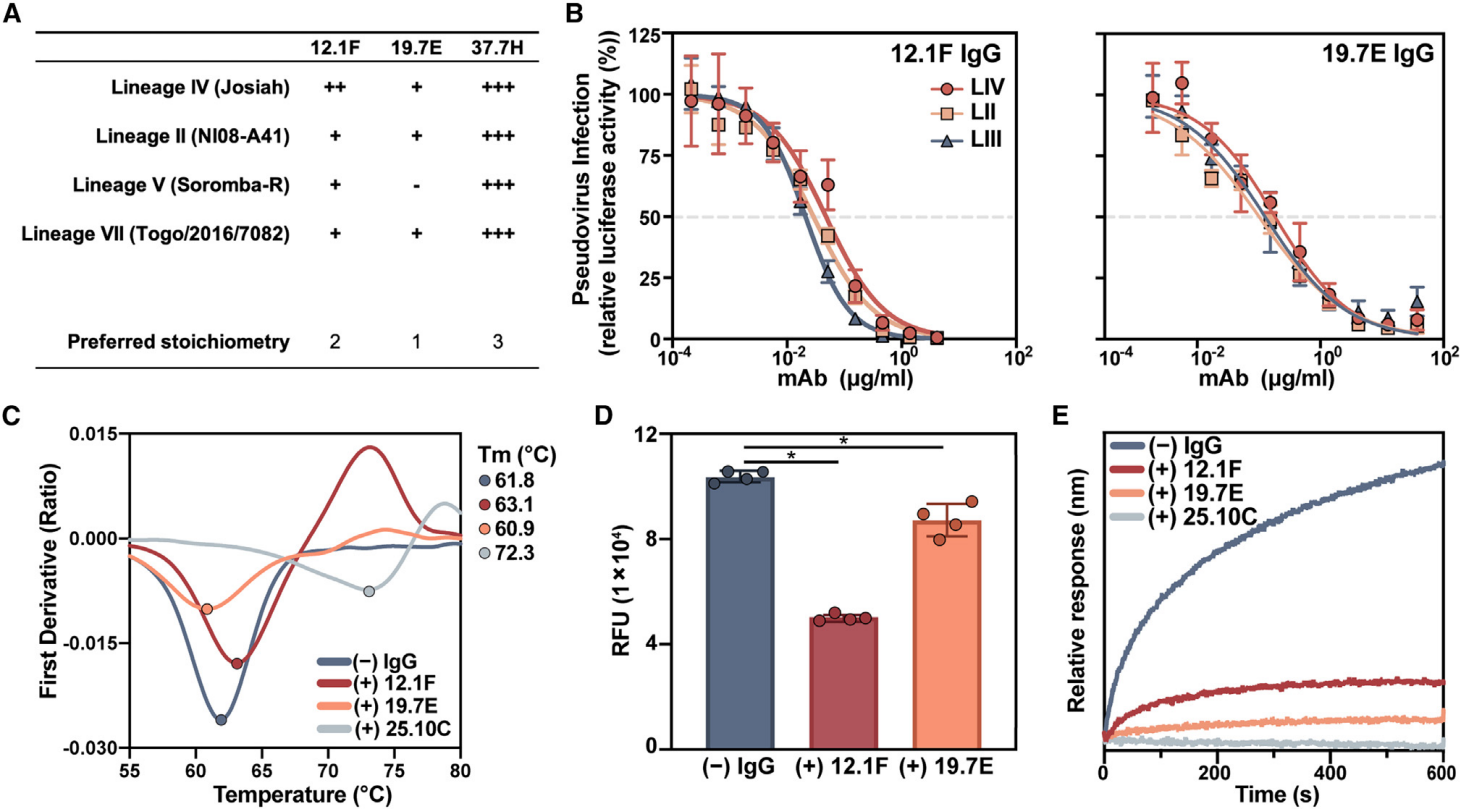
Characterization of the neutralizing GP1-A-specific mAbs 12.1F and 19.7E (A) Summary of mAb binding to GPCs by BLI (raw data in Figure S7). Binding efficiency is based on the relative on-rate of IgG to immobilized GPCs and is indicated as follows: +++, very strong binding; ++ strong binding; +, moderate binding; −, minimal binding. Proposed IgG stoichiometry per GPC is estimated based on relative R_max_ values under the assumption that the highest R_max_ indicates full occupancy and that 37.7H has a preferred occupancy of 3 Fabs per trimer, as in the crystal structure.^30^ (B) mAb neutralization of pseudoviruses derived from LASV LIV (strain Josiah), LII (strain NIG08-A41), and LIII (strain CSF). Dotted lines indicate 50% neutralization. Data points represent the mean with error bars indicating the SEM of three technical replicates. (C) Thermostability of LIV GPC-I53-50A in complex with indicated Fabs assessed by nanoDSF. Points represent the *T*_m_ of each complex. Each melting curve is a representative of triplicate curves with *T*_m_ within ±0.1°C. (D) Synthetic matriglycan competition microarray measuring StrepTagged GPC-I53-50A binding to matriglycan with and without pretreatment with 12.1F and 19.7E IgG. GPC-I53-50A bound to matriglycan was detected using StrepMAB Ab (Figure S9E). Column height reflects the mean RFU with error bars indicating standard deviation. Statistical differences between the groups (n = 4 technical replicates) were determined using two-tailed Mann-Whitney U tests (^∗^p < 0.05). (E) BLI competition analysis of immobilized GPC bound to indicated IgG and then exposed to recombinant LAMP-1 at a pH of 5 (Figure S9F). Presented data indicate representative curves from three technical replicates.

To identify differences between 12.1F and 19.7E at the phenotypic level, we analyzed the GPC binding and neutralization of these mAbs to a broad panel of LASV lineages (Figures 3A, 3B, S7, S8A, and S9A). Using our suite of stable GPC-I53-50As, we performed biolayer interferometry (BLI) experiments and observed marked differences between the binding behavior of 12.1F and 19.7E among the lineages (Figures 3A and S7). When comparing the on-rate of IgG binding to an immobilized GPC (Figure S7B), we observed that the LIV GPC-I53-50A had the highest overall binding efficiency to the tested NAbs. This finding makes sense as the LIV GPC was used as the capture antigen during mAb isolation and both patients from whom the B cells were derived were from Sierra Leone, where LIV LASV dominates.^19^^,^^64^

While 12.1F maintained binding to all GPCs tested, 19.7E showed minimal binding to LV GPC and weaker relative binding to all other lineages. Both GP1-A-targeting mAbs demonstrated a benefit from avidity effects, with both 12.1F and 19.7E showing higher dissociation rates of Fabs compared with IgGs (Figure S9A). Furthermore, we were able to estimate the apparent binding stoichiometry of these Fabs based on the proportional R_max_ values relative to 37.7H (Figure S7C)—which we assumed to bind with one Fab per protomer based on previous work (i.e., three Fabs per trimer).^42^ We showed that 12.1F and 19.7E bind with lower presumed stoichiometries of two or one Fab per trimer, respectively, and this result was further corroborated by cryo-EM for 12.1F (Figure S9B). While the majority of observed 12.1F-GPC complexes feature 2 Fabs to 1 GPC trimer under saturating conditions, 1 Fab to 1 GPC trimer and 3 Fabs to 1 GPC trimer were also observed. To assess differences in neutralization breadth, we performed pseudovirus neutralization assays. We observed neutralization of viruses pseudotyped with GPCs from LII, LIII, and LIV by both 12.1F and 19.7E, consistent with the binding data (Figure 3B). Both 12.1F and 19.7E show a heavy reliance on avidity for potent neutralization, as evidenced by a relative decrease in potency of 12.1F Fab compared with IgG and a complete lack of neutralization by 19.7E Fab (Figures S8A and S8B). The differences in Fab neutralization capabilities may result from an increased off-rate of Fabs compared with IgG, which we noted in binding studies (Figure S9A).

To assess the mechanism of binding and neutralization for these mAbs, we performed nanoDSF experiments, a matriglycan microarray competition assay,^36^ and BLI-based LAMP-1 competition experiments (Figures 3C–3E). The GPC-B-specific mAb 25.10C is known to stabilize the GPC’s prefusion conformation.^43^ Consistent with this finding, we observed that 25.10C dramatically increases the melting temperature (*T*_m_) of LIV GPC-I53-50A by >10°C. In contrast, 12.1F and 19.7E had only marginal effects on GPC thermostability, suggesting that GP1-A-specific mAbs likely do not neutralize by stabilizing the prefusion state of GPCs. To probe the interaction between the GPC and the matriglycan moieties of α-dystroglycan, we used a microarray presenting chemoenzymatically generated matriglycan oligosaccharides of a defined length.^36^ GPC-I53-50A with a furin cleavage site (GPCysR4-I53-50A) was unable to bind the matriglycan array; however, LIV GPC-I53-50A featuring the native S1P cleavage site showed potent binding (Figure S9C). Consistent with previous observations, GPC bound matriglycan in a length-dependent manner (Figure S9D).^24^^,^^36^ Whereas LIV GPC-I53-50A showed strong binding to the microarray with 24 repeating disaccharide units, the same protein complexed with 12.1F bound 51% less (Figures 3D and S9E; median relative fluorescence units [RFUs] of 1 × 10^5^ and 0.5 × 10^5^ without and with 12.1F, respectively, two-tailed Mann-Whitney U test; p = 0.029). In contrast, 19.7E showed 16% inhibition of matriglycan binding (Figures 3D and S9E; median RFUs of 1 × 10^5^ and 0.8 × 10^5^ without and with 19.7E, respectively, two-tailed Mann-Whitney U test; p = 0.029). Interestingly, the GPC-A-targeting mAb 25.10C also inhibited matriglycan binding while GPC-B mAb 37.7H did not (Figure S9E). Furthermore, both mAbs show strong inhibition of GPC binding to recombinant LAMP-1 at pH 5 with inhibition levels comparable to 25.10C, which has been shown to completely block GPC binding to LAMP-1 (Figures 3E and S9F).^43^

### Structural characterization of 12.1F and 19.7E mAbs reveals glycan dependence

**Figure 4:**
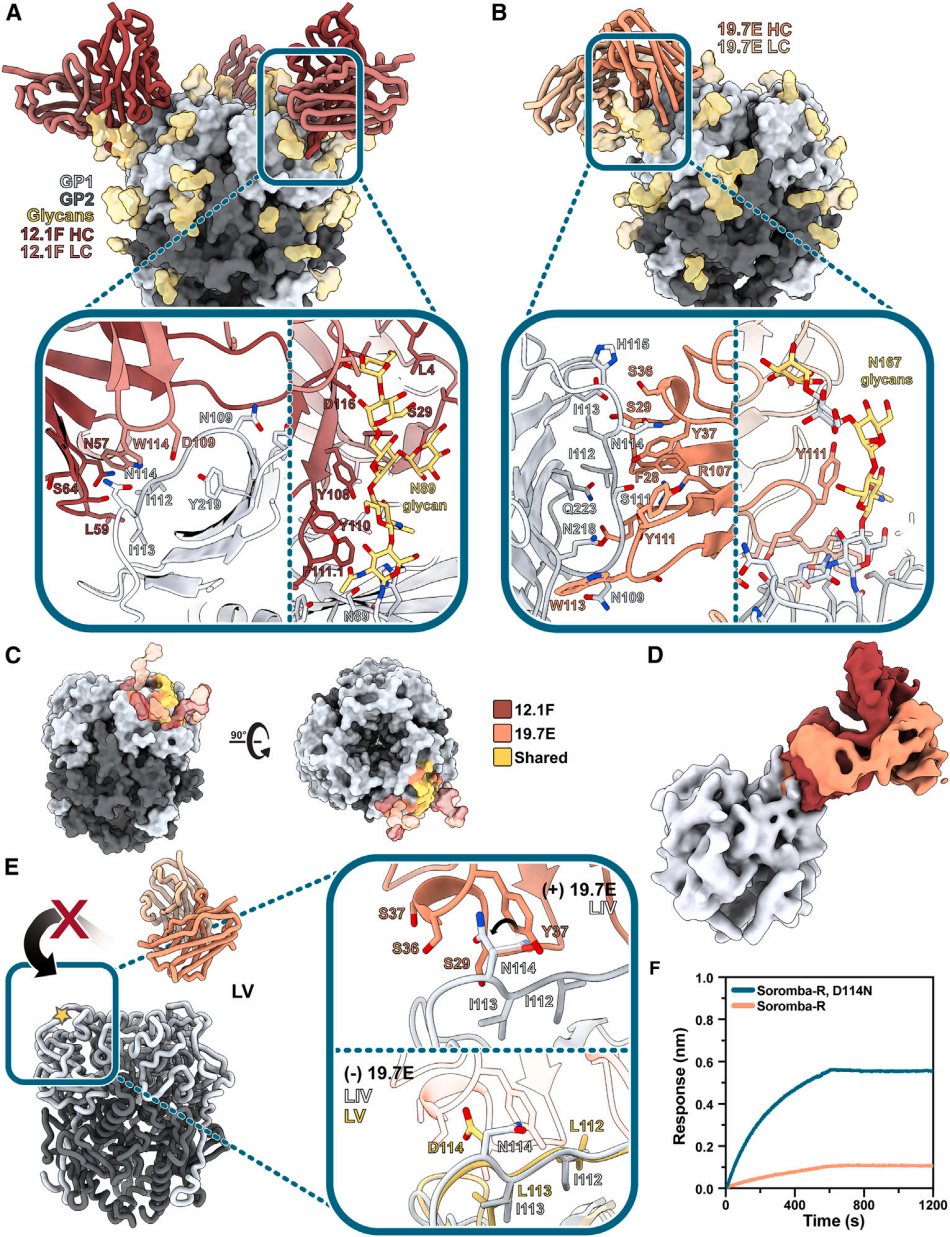
Structural description of the GP1-A epitope cluster (A) Atomic model of LIV GPC (gray) bound to 12.1F Fab (red) determined by cryo-EM. Inset depicts key interactions between GP1 and 12.1F Fab at the epitope-paratope interface. Glycans within close proximity (<4 Å) shown in gold. More details can be found in Table S2. (B) Atomic model of LIV GPC (gray) bound to 19.7E Fab (orange) determined by cryo-EM. Inset depicts key interactions between GP1 and 19.7E Fab at the epitope-paratope interface. Glycans within close proximity (<4 Å) shown in gold. More details can be found in Table S3. (C) The GP1-A antigenic landscape mapped on LIV GPC and colored according to the 12.1F (red), 19.7E (orange), or shared (yellow) Ab footprint. Glycan contacts are noted as transparent surfaces colored according to Fab interaction. (D) Overlaid, Gaussian-filtered maps showing the angle of approach taken by 12.1F (red) and 19.7E (orange) Fabs to engage LIV GPC (gray). (E) Analysis of the residues at the 19.7E binding site for LIV and LV GPCs. The gold star indicates the loop in the inset panels (right). The top panel shows the LIV GPC conformation when bound to 19.7E with the rotameric shift of LIV’s N114 shown. The bottom panels shows both LIV and LV GPCs in their unliganded conformation with 19.7E shown in translucent orange to indicate its positioning when bound to the LIV GPC. Marked residues indicate differences in the amino acid sequences of LIV and LV. (F) BLI binding analysis of immobilized 19.7E IgG binding to 140 nM of the following LV GPC-I53-50As: native strain Soromba-R (orange) or Soromba-R featuring a D114N mutation (teal; left). Presented data indicate representative curves from three technical replicates.

To assess the molecular interactions between GP1-A-targeting Abs and GPC, we used single-particle cryo-EM and determined the structures of 12.1F and 19.7E bound to the LIV GPC (Figures 4A and 4B) to 3.7 and 3.8 Å, respectively (PDB: 8EJH and 8EJI; EMDB: EMD-28182 and EMD-28183). Our models reveal that both Abs bind near the apex of the trimer, with each Fab engaging a single GP1 subunit on the loop that extends over β5–β8. 12.1F uses both its HC and LC to interact with the GPC, while 19.7E almost exclusively relies on its HC. 12.1F and 19.7E both bind in the space between apical glycans N89, N109, and N167 and show extensive contacts with the GPC glycans with total buried surface areas of 1,549 and 1,123 Å^2^, respectively.

While our previous observations and 2D classifications suggest that 12.1F typically binds in a 2 Fab-per-1 GPC fashion, applying C3 symmetry to the data enabled the best resolution of the epitope-paratope interaction (Figure 4A). Amino acid residues at the epitope-paratope site primarily interact through hydrogen bonding with the residues past the β5 sheet and before the apex-associated α1 helix, with the HC’s CDRH2 loop providing the most notable amino acid contacts (N57, L59, S64, and T65; Figure 4B, inset, left; IMGT numbering; Table S2). The LC’s CDRL3 residues predominantly engage with GP1’s S111 to contribute additional stability through hydrogen bonding. The CDRL2 sits beside the N89 glycan. The 18 amino acid CDRH3 loop of 12.1F, while in close proximity (<4 Å) to GP1, only weakly associates with GP1 amino acids. Instead, the CDRH3 makes extensive contacts with the apex glycans despite the small variability observed in Figure 2A. The HC interacts heavily with the N89 glycans, and multiple aromatic residues (Y108, Y110, F111.1; IMGT numbering) engage with the sugar moieties (Figure 4A). This trend extends to glycan N109, which interacts with W112 of the HC. The glycans we modeled contribute 59% of the total buried surface area between the Fab and GPC with individual glycan contributions of 547 (N89), 191 (N109), and 33 Å^2^ (N167). Additional contacts are described in Table S2. We observed density for the fusion peptide of GPC bound to 12.1F in two conformations: (1) similar to unliganded GPCs (Figure 2E) and (2) similar to 18.5C-, 37.7H-, 25.6A-, and 25.10C-bound GPC (Figures 2E and S6B). Importantly, our structure of 12.1F-bound GPC-I53-50A shares remarkable similarity to another 12.1F-bound GPC (PDB: 7UOV; Figure S10A)^44^ with a Cα RMSD of 0.72 Å among 512 pruned atom pairs within the GP1 subunit and Fabs and 1.8 Å across all sequence-aligned Cα pairs. Further, glycans in both structures—including the N89 glycan shown in Figure S10A—take on similar positions, strengthening our independent claims of the importance of apex glycans in the 12.1F-GPC binding event.

For 19.7E, we typically saw one Fab bound per GPC trimer and thus used symmetry-expanded particles to achieve a subset of protomers bound to the Fab. This Ab makes more contacts with amino acid residues than 12.1F (Figure 4B, left; Table S3), entirely via the HC. 19.7E engages with GP1 residues along the β-sheet surface using its CDRH1 and CDRH3 loops. Amino acid contacts of interest include GP1’s S111, which likely hydrogen bonds with HC’s Y37, R107, and/or D112. GPC residues I112 and I113 also have multiple potential hydrogen bonding partners including 19.7E's S29 and Y37. While most interactions at this interface are facilitated by hydrogen bonding, hydrophobic packing between GPC’s Y172 and W113 of the CDRH3 loop as well as GPC’s I112 with F28 and V2 of the HC also contribute to the Ab’s ability to bind GPC. While 19.7E also utilizes the apex N89, N109, and N167 glycans (Figures 4B and 4C), it shares considerably fewer interacting partners when compared with 12.1F (Tables S2 and S3). The LC only interacts minimally with the N89 and N109 glycans. The modeled GPC glycans contribute 47% of the total buried surface area when 19.7E binds to the GPC with individual glycan contributions of 251 (N167), 147 (N109), and 128 Å^2^ (N89). Upon GPC binding to 19.7E, the fusion peptide takes on a similar conformation as seen when complexed with GPC-A- and GPC-B-targeting NAbs and extends toward the trimer interior.^30^^,^^42^^,^^43^

As we noticed that the GP1-A-specific Abs shared extensive interaction networks with the apex glycans, we decided to assess whether neutralization by these mAbs is glycan dependent, as has been seen previously with the NAb LAVA01.^51^ While we observed exceptional interactions of both NAbs with the N89 glycan, previous studies indicate that N89 glycan removal leads to cleavage inefficiency. Similarly, an N109Q or N109A substitution also leads to reduced proteolytical processing.^46^ Therefore, we generated pseudoviruses containing the S111A and N167Q glycan knockout mutations. The 12.1F mAb’s neutralization potency was drastically reduced after knocking out the N109 glycan. The 19.7E mAb required both the N109 and N167 glycans to neutralize the LIV LASV pseudovirus (Figure S10B).

Inspection of the structures support the LAMP-1 and matriglycan competition we observed for these GP1-A mAbs. The 12.1F and 19.7E Fabs come within close proximity of H92 (Table S2), which—together with H93 and H230—constitutes the histidine triad and regulates the onset of pH-dependent conformational changes in GP1 required for LAMP-1 binding.^34^^,^^41^^,^^65^ While there are no additional contacts between 12.1F and 19.7E and the putative LAMP-1-binding site outside of H92 (Figure S10C), it is likely the Fabs are inhibiting LAMP-1 binding through steric hindrance or by disabling the required conformational changes. We observed an apparent discrepancy when inspecting the location of the 12.1F and 19.7E epitopes and the extent of matriglycan competition. Whereas 12.1F showed a much stronger ability to compete with matriglycan than 19.7E, the latter makes closer molecular contacts to the apex of the GPC (Figure 4E). Regardless, the interactions at both epitope-paratope interfaces do not directly interfere with residues known to associate with matriglycan (Figure S10D).^24^ The results can be reconciled by considering the angles of approach of these mAbs, as we observed the 12.1F Fab engaged at a steeper angle relative to the GPC’s 3-fold symmetry axis, which presumably causes steric impediment of matriglycan engagement. (Figure 4D). Densities of important glycans are shown in Figure S10E.

Our ligand-free structures (Figure 2) enable mapping of single-point mutations responsible for antigenic differences among LASV lineages and analysis of accompanying structural ramifications. We observe that mutations at residues 112–114 are likely to be responsible for the loss of 19.7E neutralization against LV. An overlay of the structures of unliganded LIV GPC with that of LIV in complex with 19.7E shows that N114 adjusts its side-chain orientation upon Fab binding and positions itself among three serine residues of the CDRH1 (Figure 4E). Comparison of unliganded and bound LIV GPCs shows that binding of 19.7E displaces the 112–114 residues by an average of 1.3 Å. In the unbound state, LV residues 112–114 (Figure 4E, gold star) extend further away from the β-sheet surface and would need to be displaced by an average of 2.1 Å to adopt the same conformation. Additionally, the D114 of LV GPC likely disrupts the electrostatic complementarity of N114 with the surrounding S29, S36, and S37 residues, resulting in the minimal binding of 19.7E to LV (Figure 4E, bottom). To test this, we expressed the LV GPC-I53-50A with a D114N mutation (Figure 4F, left). Indeed, the D114N mutation restored binding of LV GPC-I53-50A to immobilized 19.7E, validating our structural observations (Figure 4E) and defining the molecular determinant of Soromba-R GPC’s resistance to 19.7E binding.

### mAb S370.7 binds to the GPC-B epitope cluster and prefers trimer over monomer

**Figure 5:**
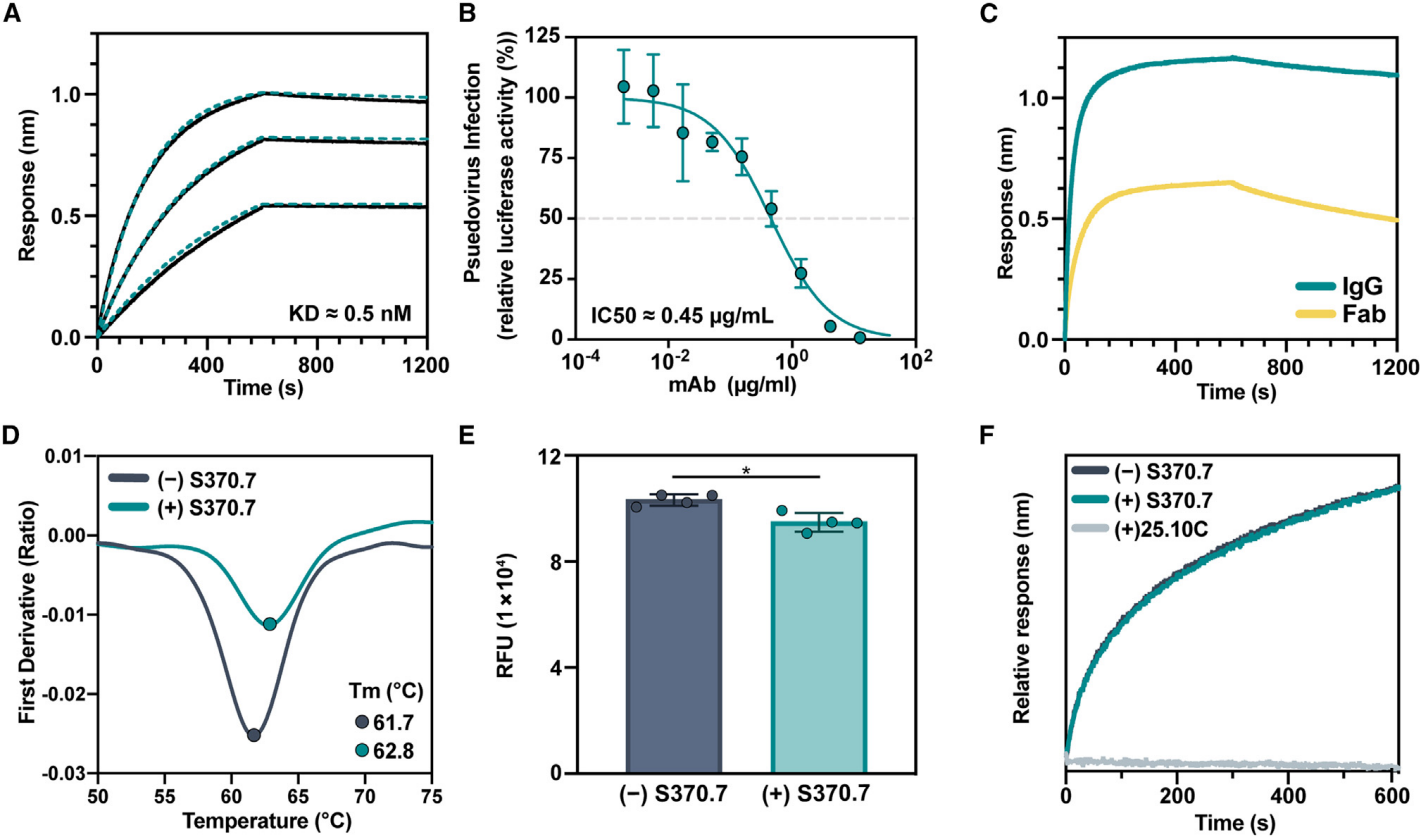
Isolation of a mAb using GPC-I53-50A (A) BLI sensorgrams depicting immobilized GPC-I53-50A binding to S370.7 IgG in a dose-dependent manner. IgG concentrations used were 50, 25, and 12.5 nM. K_D_ value determined using a 1:1 binding profile and assuming partial dissociation. Further details in Figure S11A. (B) LIV LASV pseudovirus neutralization by S370.7. Dotted line indicates 50% neutralization. Data points represent the mean with error bars indicating the SEM of three technical replicates. (C) BLI sensorgram comparing immobilized GPC binding by S370.7 IgG and Fab. IgG and Fab were added at an equimolar concentration of 400 nM. Presented data indicate representative curves from three technical replicates. (D) Thermostability of LIV GPC-I53-50A in complex with S370.7 assessed by nanoDSF. Points represent the *T*_m_. Each melting curve is a representative of triplicate curves with *T*_m_ within ±0.1°C. (E) Synthetic matriglycan binding microarray of StrepTagged GPC-I53-50A bound to S370.7 IgG and detected using StrepMAB Ab. Column height reflects the mean with error bars indicating standard deviation. Statistical differences between the groups (n = 4 technical replicates) were determined using two-tailed Mann-Whitney U tests (^∗^p < 0.05). (F) BLI analysis of immobilized GPC bound to S370.7 or 25.10C IgG and then exposed to recombinant LAMP-1 at a pH of 5. Presented data indicate representative curves from three technical replicates.

We previously showed that GPC-I53-50A proteins represent useful baits for antigen-specific B cell sorting.^51^ To expand the repertoire of available anti-GPC mAbs, we used LIV GPC-I53-50A as a bait for antigen-specific B cell sorting of convalescent serum from patient 1102370, a member of the Lassa fever survivor cohort at the Kenema Government Hospital.^19^ In doing so, we isolated a mAb, S370.7, that binds with high affinity to the GPC (Figures 5A and S11A) and neutralizes LIV pseudovirus with an IC50 of 0.45 μg/mL (Figure 5B). Similar to the GP1-A-specific Abs, S370.7 benefits from avidity, as evidenced by the increased off-rate of Fab from the GPC compared with IgG (Figure 5C). S370.7 only marginally increases the stability of the GPC-I53-50A trimer by nanoDSF, in contrast to mAbs 25.10C and 37.7H (Figures 5D and S11B). Interestingly, this mAb does not inhibit LAMP-1 binding and blocked matriglycan attachment to the GPC by only 8% (Figures 5E and 5F; median RFUs of 1 × 10^5^ and 0.9 × 10^5^ without and with S370.7, respectively, two-tailed Mann-Whitney U test; p = 0.029). Consistent with our inability to find a neutralization mechanism, S370.7 was unable to neutralize authentic Josiah virus (Figure S11C), revealing a discrepancy between HIV-based pseudovirus and authentic neutralization assays.

**Figure 6:**
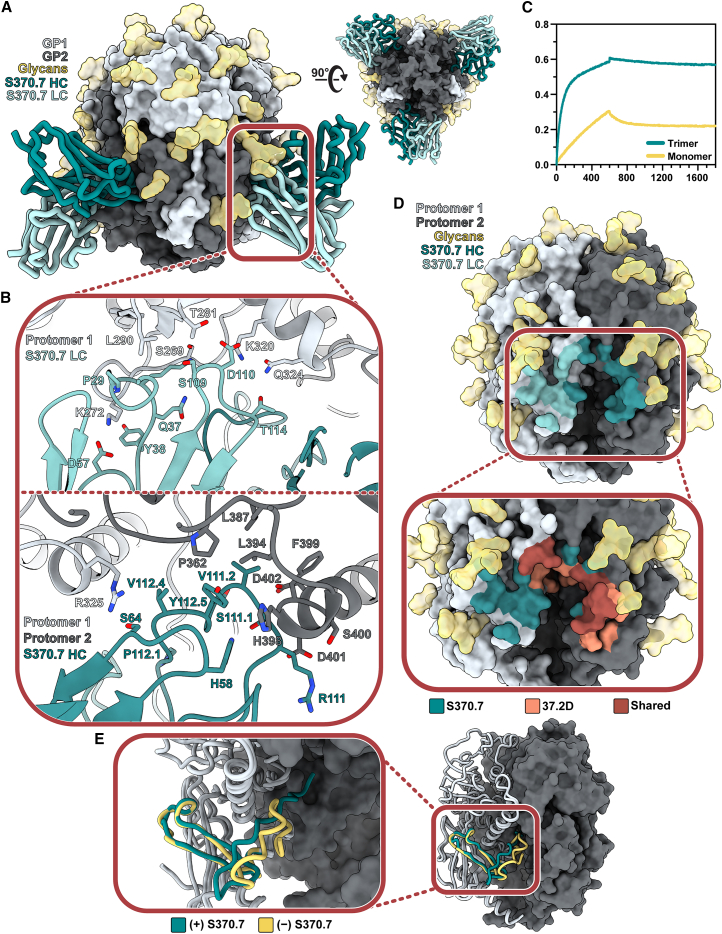
Structural characterization of the trimer-preferring mAb S370.7 (A) Atomic model of LIV GPC (gray) bound to S370.7 Fab (teal) determined by cryo-EM. (B) Key interactions between S370.7 LC (top) and HC (bottom) residues with GPC. More detailed information can be found in Table S4. (C) BLI sensorgram showing the binding profile of immobilized S370.7 IgG to GPC trimer or GPC monomer in equal protomer concentrations. Presented data indicate representative curves from three technical replicates. (D) S370.7 Ab footprint. HC interactions are shown in dark teal and LC interactions in light teal. Inset image shows the overlap and distinctions with known GPC-B-binding NAb 37.2D. Further comparisons can be drawn between Tables S4 and S5. (E) Comparison of the fusion peptides of S370.7-bound LIV GPC (teal) with unbound LIV GPC (PDB: 8EJD; yellow).

To further probe the molecular interactions between S370.7 and GPC, we determined a 3.2 Å structure of GPC-I53-50A bound to S370.7 Fab by single-particle cryo-EM (Figure 6A). The model reveals that S370.7 engages two adjacent protomers of the GPC with interactions almost exclusively within GP2. The S370.7 HC and LC primarily contact separate protomers of the GPC. The HC, which features a 22 amino acid CDRH3 loop—longer than is typical for anti-LASV Abs^19^—has a 6.5% somatic hypermutation in its IGHV4-34^∗^02 gene and penetrates the pocket situated between the fusion peptide of one protomer and the HR1d, HR2, and T-loop domains of the neighboring protomer. Both the HC and LC are flanked by the N390 and N79 glycan, respectively, with minor contacts made between each (Table S4).

The LC, which features a 3.8% somatic hypermutation rate in its IGLV3-25^∗^03 gene, engages the GPC (Figure 6B, top) with its CDRL1 (11 amino acids), CDRL2 (8 amino acids), and CDRL3 loops (11 amino acids). The CDRL1 forms hydrogen bonds with residues K272 and S269 of the GP2 fusion peptide. Additionally, K272 interacts with the CDRL2 loop, forming a salt bridge with D57. The CDRL3 loop residues D110 and T114 form hydrogen bonds with K320 and Q324 of the HR1 helix. LC's D110 and GPC's K320 likely engage further and form a salt bridge, strengthening the interaction. While the HC almost exclusively interacts with GPC via its CDRH3 (Figure 6B, bottom), its 7 amino acid CDRH2 putatively forms a hydrogen bond at S64 with R325 of GPC, making it the strongest cross-protomer interaction of the HC. Interactions between hydrophobic residues of the CDRH3 (Y112.5, V112.4, and V111.2) with residues just upstream of and extending to the HR2 helix of GP2 (L387, S389, L394, and F399) support Ab binding by forming a stable, hydrophobic pocket. Just beyond this hydrophobic pocket, there appears to be an additional favorable electrostatic interaction forming between D401 of GP2 and R111 of the CDRH3. The total buried surface area between the Fab and GPC is 1,275 Å^2^, of which the modeled glycans contribute 22%.

Based on the nature of the S370.7 epitope, we hypothesized that S370.7 requires the correct quaternary presentation of GPC for efficient binding. We found that while S370.7 could still bind LIV GP monomer, it did so at a reduced rate with higher dissociation compared with its binding to the LIV GPC trimer, making the S370.7 mAb trimer preferring (Figure 6C). Compared with other known Abs (Figure S11D), S370.7 exhibits the highest degree of preference for the trimeric conformation of GPC. We compared the epitopes of S370.7 and 37.2D, a member of the Arevirumab-3 mAb cocktail,^44^^,^^45^ and observed a substantial overlap, especially within the region upstream of the HR2 helix, implying that S370.7 is a member of the GPC-B competition group of anti-LASV mAbs (Figures 6D and S11E; Tables S4 and S5). We also note a conformational change in the fusion peptide upon binding to S370.7 (Figure 6E), which is consistent with our observations of other Ab-bound fusion peptide conformational differences (Figures 2E and S6B).

## Discussion

The advancement of prefusion-stabilized GPCs is an important step for developing useful immunogens and reagents to study the humoral immune response to LASV and LASV vaccines.^66^^,^^67^ Here, we further demonstrate the use of the I53-50A protein as a trimerization scaffold for the stabilization of GPCs of four of the seven currently proposed LASV lineages. The GPC-I53-50As are a suite of stable, soluble heterologous proteins useful for assessing cross-binding of mAbs and are amenable to cryo-EM analysis when high-resolution information is needed. Importantly, the GPC-I53-50As present native-like epitopes and bind to Abs within the canonical GP1-A, GPC-A, and GPC-B competition groups without the need for additional stabilizing Abs. Our ligand-free GPCs enable tracking of the fusion peptide response to Ab binding, thus enabling more complete insights into the binding and neutralization mechanisms of anti-GPC Abs.

The GPC structures bound to 12.1F and 19.7E presented here define the GP1-A competition group and show that their epitope resides near the apex of the GP1 protomer and interacts widely with apical glycans. Glycan dependence is confirmed through glycan knockout pseudovirus neutralization assays and through recently published, complementary work.^44^ These Abs contribute to LASV neutralization by hindering GPC’s ability to (1) bind the matriglycan sugars of its extracellular receptor α-dystroglycan and (2) engage with the endosomal receptor LAMP-1. Intriguingly, we also observed GPC-A-targeting mAb 25.10C inhibits matriglycan binding despite its epitope residing near the middle of the GPC.^43^ The inhibition of GPC binding to both matriglycan and LAMP-1 by 12.1F and 25.10C mAbs may explain their more potent neutralizing properties compared with other isolated mAbs, especially in light of findings that LAMP-1 is not necessary for LASV fusion.^19^^,^^68^^,^^69^^,^^70^

Finally, we demonstrate that GPC-I53-50As are valuable baits for antigen-specific human B cell sorting with our discovery of GPC-B-targeting Ab S370.7 using LIV GPC-I53-50A. This mAb engages the GPC in a similar fashion as the majority of known neutralizing anti-GPC Abs and uses both its HC and LC, which are flanked by N79 and N390 glycans, to engage adjacent protomers. Binding by S370.7 causes migration of the fusion peptide to the interior of the trimer, where it resides beneath the C-terminal of GP2. While initial pseudovirus assays repeatedly showed neutralization of LIV virus by S370.7, an authentic virus neutralization assay demonstrated a lack of neutralizing ability by S370.7 within the concentrations tested. These findings demonstrate the importance of using authentic virus assays as the gold standard to assess the neutralizing efficacy of new mAbs for their use as potential therapeutics, though weakly neutralizing mAbs may assist in virus clearance via Fc-mediated effector functions and can be valuable as reagents for the field.

The success of the GPC-I53-50A antigens for identifying previously unknown Abs has implications beyond the scope of this work. As has been well evidenced by the SARS-CoV-2 pandemic, viral pathogens are likely to escape mAb treatment as they evolve. While the Arevirumab-3 mAb cocktail shows incredible promise as a treatment for Lassa fever,^44^^,^^45^ we must account for a potential loss in efficacy if Lassa continues to evolve by immune pressures.^71^ As our approach describes a trimerization mechanism that accommodates multiple strains of LASV GPC across lineages and can be modified quickly with minimal protein engineering, it can be adapted for use with new strains of concern. Further, applying this stabilization scheme to additional arenaviruses presents the exciting opportunity to screen for Abs capable of binding across Old and New World arenaviruses.

In summary, our findings and the suite of I53-50A stabilized GPC ectodomains (1) describe stable, trimeric GPC reagents for cross-binding assessment, (2) provide a robust and relatively high-throughput platform for single-particle cryo-EM analysis of LASV GPCs with and without mAbs, (3) inform more comprehensive immunogen design and stabilization work, specifically in the context of GP1-A-targeting Abs, and (4) allow for B cell sorting of strain-specific and broadly reactive mAbs.

### Limitations of the study

While our GPC-I53-50A proteins share high structural similarity to full-length LASV GPCs (Figure S3A), it is important to note that our constructs do not retain the SSP, which plays an important role in GPC processing and is a critical component of the mature GPC on the viral surface.^72^^,^^73^ While no known Abs engage the SSP, it is possible that its absence results in the loss of a neutralizing epitope or the addition of a neo-epitope, which may result in the induction of non-neutralizing responses if GPC-I53-50A is used as an immunogen.

We note that 12.1F inhibits matriglycan binding by 51% despite not overlapping with the known matriglycan binding site. At this time, the exact molecular mechanism by which these GP1-A-specific Abs affect receptor binding is unknown. Further studies are needed to define the exact mechanisms.

## STAR★Methods

### Key resources table

**Table:** 

REAGENT or RESOURCE	SOURCE	IDENTIFIER
**Antibodies**		

19.7E	Robinson et al., 2016	Patent WO2018106712A1
12.1F	Robinson et al., 2016	Patent WO2018106712A1
25.10C	Robinson et al., 2016	Patent WO2018106712A1
37.7H	Robinson et al., 2016	Patent WO2018106712A1
S370.7	This study	N/A
StrepMAB-Classic Oyster 645 conjugate	IBA Lifesciences	Cat# 2-1555-050
Cy3 conjugated goat-anti-human	Jackson Immuno Research	Cat# 109-165-008; RRID: AB_2337720
Totalseq-C Anti-Human Hashtag #1	Biolegend	Cat# 394661; RRID: AB_2801031
Totalseq-C Anti-Human Hashtag #2	Biolegend	Cat# 394663; RRID: AB_2801032
Totalseq-C Anti-Human Hashtag #3	Biolegend	Cat# 394665; RRID: 2801033
Totalseq-C Anti-Human Hashtag #4	Biolegend	Cat# 394667; RRID: 2801034
APC-Cy7 Mouse Anti-Human CD3	BD Biosciences	Cat# 557757; RRID: AB_396863
APC-Cy7 Mouse Anti-Human CD4	Biolegend	Cat# 317418; RRID: AB_571947
APC-Cy7 Mouse Anti-Human CD8	BD Biosciences	Cat# 557760; RRID: AB_396865
APC-H7 Mouse Anti-Human CD14	BD Biosciences	Cat# 561384; RRID: AB_10611720
PerCP-Cy5.5 Mouse Anti-Human CD19	Biolegend	Cat# 302230; RRID: AB_2073119

**Bacterial and Virus Strains**		

NEB 5-alpha Competent E. coli (High Efficiency)	New England Biolabs	Cat# C2987H
Lassa virus (Josiah strain)	Brouwer et al., 2022	N/A

**Biological Samples**		

Convalescent patient serum	Robinson et al., 2016	N/A

**Chemicals, Peptides, and Recombinant Proteins**		

Tween20	Sigma-Aldrich	Cat# P1379-500ML
Bovine Serum Albumin (fraction V)	Thermo Scientific	Cat# 9048-46-8
Penicillin	Sigma-Aldrich	Cat# P3032-10MI
Streptomycin	VWR	Cat# 382-EU-100G
Iodacetamide	Sigma-Aldrich	Cat# I1149
BioLock solution	IBA Lifesciences	Cat# 2-0205-050
PBS	Thermo Scientific	Cat# 10010023
TBS	Alfa Aesar	Cat# J60764.K2
PEI MAX	Polysciences	Cat# 24765-1
Uranyl Formate	Electron Microscopy Sciences	Cat #D310 25 GM
Fluorinated octyl maltoside	Anatrace	Part# O310F
Imidazole	Sigma-Aldrich	Cat# I5513
Papain from papaya latex	Sigma-Aldrich	SKU# P4762
Acetonitrile, 80%, 20% Water with 0.1% Formic Acid, Optima LC/MS	Fisher Scientific	Cat# 15431423
Water with 0.1% Formic Acid (v/v), Optima™ LC/MS Grade	Fisher Scientific	Cat# LS118-212
Acetonitrile	Fisher Scientific	Cat# 10489553
Trifluoroacetic acid	Fisher Scientific	Cat# 10155347
Dithiothreitol	Sigma-Aldrich	Cat# 43819
Mass spectrometry grade trypsin	Promega	Cat# V5280
Sequencing grade chymotrypsin	Promega	Cat# V1061
α-Lytic Protease	New England Biolabs	Cat# P8113L
Urea	Sigma-Aldrich	U5378-1KG
Fugene	Promega	Cat# E5911
DEAE-dextran	Sigma-Aldrich	Cat# D9885
Saquinavir	NIH-ARP	Cat# 4658
Reporter Lysis buffer	Promega	Cat# E3971
Fetal Bovine Serum	Omega Scientific, Inc.	FB-02
RPMI 1640	Corning	15-040-CV
Trypan Blue	Sigma-Aldrich	T8154-100ML
Ethanol	Pharmaco	111000200
FectoPRO	Polyplus Transfection	101000019
Citric acid	Fisher Scientific	BP339-500
Tris Base	Sigma-Aldrich	T1503-5KG

**Critical Commercial Assays**		

Bright-Glo Luciferase Assay System	Promega	Cat# E2620

**Deposited Data**		

3D map of LIV GPC-I53-50A (strain Josiah)	This study	EMDB: EMD-28178
3D model of LIV GPC-I53-50A (strain Josiah)	This study	PDB: 8EJD
3D map of LII GPC-I53-50A (strain NIG08-A41)	This study	EMDB: EMD-28179
3D model of LII GPC-I53-50A (strain NIG08-A41)	This study	PDB: 8EJE
3D map of LV GPC-I53-50A (strain Soromba-R)	This study	EMDB: EMD-28180
3D model of LV GPC-I53-50A (strain Soromba-R)	This study	PDB: 8EJF
3D map of LVII GPC-I53-50A (strain Soromba-R)	This study	EMDB: EMD-28181
3D model of LVII GPC-I53-50A (strain Soromba-R)	This study	PDB: 8EJG
3D map of LIV GPC-I53-50A (strain Josiah_ bound to 12.1F Fab	This study	EMDB: EMD-28182
3D model of LIV GPC-I53-50A (strain Josiah_ bound to 12.1F Fab	This study	PDB: 8EJH
3D map of LIV GPC-I53-50A (strain Josiah_ bound to 19.7E Fab	This study	EMDB: EMD-28183
3D model of LIV GPC-I53-50A (strain Josiah_ bound to 19.7E Fab	This study	PDB: 8EJI
3D map of LIV GPC-I53-50A (strain Josiah_ bound to S370.7 Fab	This study	EMDB: EMD-28184
3D model of LIV GPC-I53-50A (strain Josiah_ bound to S370.7 Fab	This study	PDB: 8EJJ
Mass spectrometry data	This study	ftp://massive.ucsd.edu/MSV000091003/
S370.7 HC sequence	This study	GenBank: OQ451467
S370.7 LC sequence	This study	GenBank: OQ451468

**Experimental Models: Cell Lines**		

FreeStyle 293F cells	Thermo Scientific	Cat# R79007
HEK 293T cells	ATCC	Cat# CRL-11268
Expi293T cells	ATCC	CRL-3216
TZM-bl cells	NIH ARRRP	Cat# 8129

**Experimental Models: Organisms/Strains**		

**Recombinant DNA**		

GPCysR4(Josiah)-StreptagII pPPI4 plasmid	Brouwer et al., 2022	N/A
GPCysR4(Josiah)-I53-50A.1NT1-Strep-tagII pPPI4 plasmid	Brouwer et al., 2022	N/A
GPCysR4(NIG08-A41)-I53-50A.1NT1-Strep-tagII pPPI4 plasmid	This study	N/A
GPCysR4(Soromba-R)-I53-50A.1NT1-Strep-tagII pPPI4 plasmid	This study	N/A
GPCysR4(Togo)-I53-50A.1NT1-Strep-tagII pPPI4 plasmid	This study	N/A
GPCysR4(Josiah)-I53-50A.1NT1-Avi-His pPPI4 plasmid	Brouwer et al., 2022	N/A
GPCysR4(NIG08-A41)-I53-50A.1NT1-Avi-His pPPI4 plasmid	Brouwer et al., 2022	N/A
GPCysR4(Soromba-R)-I53-50A.1NT1-Avi-His pPPI4 plasmid	This study	N/A
GPCysR4(Togo)-I53-50A.1NT1-Avi-His pPPI4 plasmid	This study	N/A
GPCysR4(Soromba-R_D114N)-I53-50A.1NT1-StrepTagII pPPI4 plasmid	This study	N/A
Furin pPPI4 plasmid	Brouwer et al., 2019	N/A
S1P pcDNA3.0 plasmid	This study	N/A
GPCysRRLL(Josiah)-I53-50A.1NT1-Strep-tagII pPPI4 plasmid	This study	N/A
Fc-tagged LAMP-1 ectodomain	Jae et al., 2014	N/A
GPC(NIG08-A41)_full-length pPPI4 plasmid	Brouwer et al., 2022	N/A
GPC(Josiah)_full-length pPPI4 plasmid	Brouwer et al., 2022	N/A
GPC(CSF)_full-length pPPI4 plasmid	Brouwer et al., 2022	N/A
19.7E HC, 19.7E LC, 12.1F HC, 12.1F LC, 25.10C HC, 25.10C LC, 37.7H HC, 37.7H LC gene fragments	Integrated DNA Technologies	N/A
S370.7 HC and LC gene fragments	This study	N/A

**Software and Algorithms**		

GraphPad Prism v8	GraphPad	N/A
UCSF Chimera	Pettersen et al., 2004	N/A
UCSF ChimeraX	Goddard et al., 2018	N/A
cryoSPARC.v3	Punjani et al., 2017	N/A
Leginon	Suloway et al., 2005	N/A
MotionCor2	Zheng et al., 2017	N/A
GCTF	Zhang, 2016	N/A
Relion/3.0 and 3.1	Zivanov et al., 2018	N/A
ABodyBuilder	Leem et al., 2016	N/A
Coot	Casañal et al., 2020	N/A
Phenix	Liebschner, et al., 2019	N/A
EMRinger	Barad et al., 2015	N/A
MolProbity	Williams et al., 2018	N/A
Appion	Lander et al., 2009	N/A
Epitope-Analyzer	Montiel-Garcia, et al., 2022	N/A
PDBePISA	Krissinel et al., 2007	N/A
Privateer	Agirre et al., 2015	N/A
Byos™ (Version 4.0)	Protein Metrics Inc.	N/A
XCalibur Version v4.2	Thermo Fisher	N/A
Orbitrap Fusion Tune application v3.1	Thermo Fisher	N/A
Cellranger 6.0.2	10X Genomics	N/A
ab[x] toolkit	Briney et al., 2018	N/A
Single Cell Analysis of B cells (SCAB)	Hurtado et al., 2022	N/A
GenePix Pro 7 software v7.2.29.2	Molecular Devices	N/A

**Other**		

PstI-HF	New England Biolabs	Cat# R3140S
BamHI-HF	New England Biolabs	Cat# R3136S
NotI-HF	New England Biolabs	Cat# R3189
Q5 Site-directed mutagenesis kit	New England Biolabs	Cat# E0554S
T4 DNA ligase	New England Biolabs	Cat# M0202
Quick ligation kit	New England Biolabs	Cat# M2200
Superdex200 10/300GL Column	GE Healthcare Life Sciences	Cat# 28990944
Protein G resin	Cytiva	Cat# 17061802
CaptureSelect IgG-Fc resin	Thermo Scientific	Cat# 2942852010
Ni-NTA agarose	QIAGEN	Cat# 30210
Amicon® Ultra-4 Centrifugal Filter Unit (100 kDA MWCO)	Millipore Sigma	SKU# UFC810024
Amicon® Ultra-4 Centrifugal Filter Unit (30 kDA MWCO)	Millipore Sigma	SKU# UFC803024
Amicon® Ultra-4 Centrifugal Filter Unit (10 kDA MWCO)	Millipore Sigma	SKU# UFC801024
Amicon Ultra-0.5 Centrifugal Filter Unit (100 kDa MWCO)	Millipore Sigma	SKU# UFC5100BK
Amicon Ultra-0.5 Centrifugal Filter Unit (30 kDa MWCO)	Millipore Sigma	SKU # UFC5030BK
Amicon Ultra-0.5 Centrifugal Filter Unit (10 kDA MWCO)	Millipore Sigma	SKU# UFC5010BK
Prometheus NT.Plex nanoDSF Grade High Sensitivity Capillary Chips	Nanotemper	Cat# PR-AC006
400-mesh copper grids	Electron Microscopy Sciences	Cat# 0400-Cu
Octet Red96 system	Sartorius (FortéBio)	N/A
Octet Biosensors: Streptavidin (SA)	Sartorius (FortéBio)	Cat# 18-5019
Octet Biosensors: Anti-Human Fc Capture (AHC)	Sartorius (FortéBio)	Cat# 18-5060
FreeStyle 293 Expression medium	Thermo Scientific	Cat# 12338018
OptiMEM	Gibco	Cat# 31985-070
DMEM	Gibco	Cat# 21969-035
Fetal calf serum	Gibco	Cat# 10270/106
BioLock	IBA Lifesciences	Cat# 2-0205-250
BXT Buffer (10X)	IBA Lifesciences	Cat# 2-1042-025
Steritop Filter Units	Merck Millipore	Cat# C3239
Greiner CELLSTAR® 96 well plates round bottom clear wells	Merck Millipore	Cat# M9436
Totalseq-C0951 PE-Streptavidin	Biolegend	Cat# 405183
Totalseq-C0992 PE-Streptavidin	Biolegend	Cat# 405181
Totalseq-C0994 PE-Streptavidin	Biolegend	Cat# 405177
Totalseq-C0956 APC-Streptavidin	Biolegend	Cat# 405261
Totalseq-C0958 APC-Streptavidin	Biolegend	Cat# 405293
Totalseq-C0971 Streptavidin	Biolegend	Cat# 405271
Totalseq-C0972 Streptavidin	Biolegend	Cat# 405273
Totalseq-C0973 Streptavidin	Biolegend	Cat# 405275
LIVE/DEAD Fixable Aqua Dead Cell Stain Kit	Thermo Scientific	Cat# L34966
Biotinylated Human Serum Albumin Protein	Acro Biosystems	Cat# HSA-H82E3
Chromium Next GEM Chip K Single Cell Kit	10X Genomics	PN# 1000287
Chromium Single Cell V(D)J Amplification Kit, Human BCR	10X Genomics	PN# 1000255
Chromium 5’ Feature Barcode Kit	10X Genomics	PN# 1000256
Chromium Next GEM Single Cell 5’ Gel Bead Kit v3	10X Genomics	PN# 1000264
Chromium Next GEM Single Cell 5’ Library Construction Kit	10X Genomics	PN# 1000190
96-well Skirted PCR plates White Barcoded	Bio-Rad	Cat# HSP9901
FACSMelody Cell sorter	BD Biosciences	N/A
Chromium Controller	10X Genomics	N/A
NEBuilder HiFi DNA Assembly Master Mix	New England Biolabs	E2621X
NovaSeq 6000 Sequencing System	Illumina	N/A
NovaSeq 6000 S4 Reagent Kit v1.5	Illumina	20028313
NovaSeq 6000 S2 Reagent Kit v1.5	Illumina	20028316
NovaSeq 6000 S1 Reagent Kit v1.5	Illumina	20028319
NovaSeq 6000 SP Reagent Kit v1.5	Illumina	20028401
Glomax reader	Turner BioSystems	Model# 9101-002
UltrAuFoil R 1.2/1.3 grids (300-mesh)	Quantifoil Micro Tools GmbH	N/A
Strep-TactinXT Superflow high capacity resin	IBA Life Sciences	Cat# 2-4010-010
NanoDrop 2000C	Thermo Scientific	Cat# ND-2000C
Prometheus NT.48 NanoDSF	NanoTemper Technologies	N/A
Leica DMi1 Inverted Microscope	Leica Microsystems	N/A
Tecnai F20 electron microscope	FEI	N/A
TemCam F415 CMOS camera	TVIPS	N/A
Vitrobot mark IV	Thermo Scientific	N/A
Solarus 950 plasma system	Gatan	N/A
PELCO easiGlow	Ted Pella Inc.	N/A
Talos Arctica	Thermo Scientific	
FEI Titan Krios	Thermo Scientific	N/A
K2 Summit direct electron detector camera	Gatan	N/A
C18 ZipTip	Merck Milipore	Cat# ZTC18S008
Vivaspin 500, 3 kDa MWCO, Polyethersulfone	Sigma-Aldrich	Cat# GE28-9322-18
Orbitrap Eclipse mass spectrometer	Thermo Fisher Scientific	N/A
Ultimate 3000 HPLC	Thermo Fisher Scientific	N/A
EasySpray PepMap RSLC C18 column (75 μm x 75 cm)	Thermo Fisher Scientific	Cat# ES805
PepMap 100 C18 3μM 75μM x 2cm	Thermo Fisher Scientific	Cat# 164946
NEXTERION Slide H NHS-ester activated glass slides	Schott Inc.	SKU:1070936
sciFLEXARRAYER S3 non-contact microarray	Scienion Inc.	N/A
Scienion PDC80 nozzle	Scienion Inc.	N/A
GenePix 4000B microarray scanner	Molecular Devices	N/A

### Resource availability

#### Lead contact

Further information and requests for resources and reagents should be directed to and will be fulfilled by the lead contact, Andrew B. Ward (andrew@scripps.edu).

#### Materials availability

All reagents will be made available on request after completion of a Materials Transfer Agreement.

### Experimental model and subject details

#### Cell lines

FreeStyle 293-F cells were purchased from Thermo Fisher Scientific. The cells were used following manufacturer protocols with details described below.

### Method details

#### Sequence alignment and conservation assessment

S genomes of LASV field isolates^56^ were aligned, matched to groups according to codon reading frame, and re-aligned based on amino acid residue using Clustal Omega multiple sequence alignment.^90^ A total of 361 GPC sequences were analyzed. Conservation was estimated using AL2CO^91^ entropy measure with the modified Henikoff & Henikoff frequency estimation method and a gap fraction of 0.7 and visualized in ChimeraX.^75^

#### Construct design

The LIV GPC monomer, LIV GPC-I53-50A, and Avi-his-tagged LIV GPC-I53-50A constructs were generated as described previously.^51^ To generate the NIG08-A41, Soromba-R, and Togo/2016/7082-GPC-I53-50A constructs, genes encoding GPC residues 1–423(GenBank: ADU56626.1), 1–424 (GenBank: AHC95553.1), and 1–423 (GenBank: AMR44577.1), respectively, with the GPCysR4 mutations introduced^30^ were cloned by Gibson assembly into PstI-BamHI-digested Josiah-GPC-I53-50A plasmid. A LIV GPC-I53-50A construct with the native S1P cleavage site was generated by introducing R258L and R259L mutations by Q5 site-directed mutagenesis. The 12.1F, 19.7E, 37.7H, and 25.10C sequences were derived from patent WIPO: WO2018106712A1. The 19.7E, 37.7H, 12.1F, 25.10C, and S370.7 plasmids were generated by Gibson assembly of genes encoding the variable regions of the corresponding heavy and light chains into plasmids containing the constant regions of the human IgG1 for the heavy or light chain. Plasmids encoding histidine-tagged Fab regions of 12.1F, S370.7, and 25.10C were generated by introducing a histidine-tag followed by a stop-codon in the hinge region (directly upstream of the DKTHT motif) of the corresponding heavy chain plasmid by Q5 site-directed mutagenesis. For pseudovirus neutralization assays, a pPPI4 plasmid was digested with PstI-NotI and a gene encoding full-length GPC of lineage II (NIG08-A41), lineage III (CSF; GenBank: AAL13212.1), or lineage V (Soromba-R) was inserted by Gibson assembly. Q5 site-directed mutagenesis was used to introduce the S111A and N167Q mutations into a plasmid encoding full-length, native Josiah GPC (a kind gift from Robin Shattock).

#### Protein expression and purification

GPC-I53-50As, LIV GPC monomer, biotinylated GPC-I53-50As and Abs were transiently expressed in HEK 293F cells at a density of 1.0 x 10^6^ cells/mL using PEImax at a ratio of 1:3 DNA to PEI. HEK 293F cells were maintained in HEK 293F in 293FreeStyle expression medium (Life Technologies) and cultured at 37°C with 8% CO_2_ while shaking at 125 rpm. Plasmids encoding GPCs were co-transfected with a furin plasmid at a 1:2 ratio. To express biotinylated GPC-I53-50A, HEK 293F cells were co-transfected with Avi-his-tagged GPC-I53-50A, furin and a BirA plasmid (a kind gift from Lars Hangartner) in a 2:1:0.5 ratio. IgG plasmids were transfected at a heavy and light chain ratio of 1:1 while the generation of Fabs of 12.1F, 25.10C, and S370.7 was initiated by transfecting the histidine-tagged heavy chain Fab domain with the corresponding light chain at a ratio of 1:2. Culture supernatants of GPC constructs were harvested after six days, while IgG and Fab were harvested after five days. GPC-I53-50As were purified by gravity column using StrepTactin 4Flow resin (IBA Life Sciences) according to manufacturer's protocol and eluted with 1X BXT (IBA Life Sciences). IgGs were purified by gravity column using Protein G (Cytiva) or CaptureSelect IgG-Fc resin (Thermo Scientific) and eluted with 0.1 M glycine at pH 2.0. Biotinylated GPC-I53-50As and Fabs of 12.1F and S370.7 were purified by rolling the culture supernatant overnight at 4°C with Ni-NTA Agarose resin (Thermo Scientific). The next day, the bead suspension was flown over a gravity column, washed with 20 mM imidazole, 50 mM NaCl, pH 7.0 and eluted with 500 mM imidazole, 50 mM NaCl buffer, pH 7.0. Recombinant LAMP-1 was generated by transfecting HEK 293F cells with a rabbit Fc-tagged LAMP-1 plasmid encoding residues A29-S351 (a kind gift from Thijn Brummelkamp).^38^ Culture supernatant was then incubated with CaptureSelect IgG-Fc resin (Thermo Scientific) and eluted from the resin using 0.1 M glycine, pH 3.0, into neutralization buffer (1 M Tris, pH 8.0) at a 1:9 ratio. All proteins were buffer exchanged to TBS after elution and purified further by size exclusion chromatography using a Superdex 200 increase 10/300 GL column (Sigma-Aldrich) with TBS as its running buffer. Fractions corresponding to the appropriate peaks were concentrated using a MWCO concentrator with the following cutoffs: 100 kDa for GPC-I53-50As; 30 kDa for IgGs and LIV GPC monomer; and 10 kDa for Fabs (Millipore).

#### Differential scanning fluorimetry

Thermostability of GPC and GPC-Fab complexes was determined with a nano-DSF NT.48 (Prometheus). GPC proteins or complexes were diluted to 0.5 mg/mL and loaded into high sensitivity capillaries. The assay was run with a linear scan rate of 1°C/min and 80%-100% excitation power. The first derivative of the ratio of tryptophan fluorescence wavelength emissions at 350 and 330 nM were analyzed to determine thermal onset (*T*_*onset*_) and denaturation (*T*_*m*_) temperatures using the Prometheus NT software.

#### Negative stain electron microscopy

Carbon-coated 400-mesh copper grids were glow discharged for 25 s at 15 mA using a PELCO easiGlow instrument (Ted Pella, Inc.). GPC-I53-50A samples were diluted in TBS to approximately 15 μg/mL and loaded onto the copper grids and incubated for 30 s. The sample was blotted and immediately stained with 2% (w/v) uranyl formate for 15 s. Excess stain was removed by blotting and grids were dried for >5 minutes before being loaded on a 200 kV Tecnai F20 electron microscope (FEI) featuring a TemCam F416 CMOS camera (TVIPS). Images were collected at a magnification of 62,000X with a defocus value of -1.5 um, total electron dose of 25 e^-^/Å^2^, and pixel size of 1.77 Å. Images were acquired using the Leginon software package.^51^ Approximately 100,000 particles were picked using Appion^86^ and 2D classification was performed with Relion 3.0.^81^

#### Site-specific glycan analysis

100 μg aliquots of each sample were denatured for 1h in 50 mM Tris/HCl, pH 8.0 containing 6 M of urea and 5 mM dithiothreitol (DTT). Next, GPC-I53-50A samples were reduced and alkylated by adding 20 mM iodoacetamide (IAA) and incubated for 1h in the dark, followed by a 1h incubation with 20 mM DTT to eliminate residual IAA. The alkylated GPC-I53-50A samples were buffer exchanged into 50 mM Tris/HCl, pH 8.0 using Vivaspin columns (3 kDa) and two of the aliquots were digested separately overnight using chymotrypsin (Mass Spectrometry Grade, Promega) or alpha lytic protease (New England Biolabs) at a ratio of 1:30 (w/w). The next day, the peptides were dried and extracted using C18 Zip-tip (MerckMilipore). The peptides were dried again, re-suspended in 0.1% formic acid and analyzed by nanoLC-ESI MS with an Ultimate 3000 HPLC (Thermo Fisher Scientific) system coupled to an Orbitrap Eclipse mass spectrometer (Thermo Fisher Scientific) using stepped higher energy collision-induced dissociation (HCD) fragmentation. Peptides were separated using an EasySpray PepMap RSLC C18 column (75 μm × 75 cm). A trapping column (PepMap 100 C18 3 μM 75 μM x 2cm) was used in line with the LC prior to separation with the analytical column. The LC conditions were as follows: 280 minute linear gradient consisting of 4-32% acetonitrile in 0.1% formic acid over 260 minutes followed by 20 minutes of alternating 76% acetonitrile in 0.1% formic acid and 4% ACN in 0.1% formic acid, used to ensure all the sample had eluted from the column. The flow rate was set to 200 nL/min. The spray voltage was set to 2.7 kV and the temperature of the heated capillary was set to 40°C. The ion transfer tube temperature was set to 275°C. The scan range was 375−1500 m/z. Stepped HCD collision energy was set to 15, 25 and 45% and the MS2 for each energy was combined. Precursor and fragment detection were performed using an Orbitrap at a resolution MS1=120,000. MS2=30,000. The AGC target for MS1 was set to standard and injection time set to auto which involves the system setting the two parameters to maximize sensitivity while maintaining cycle time. Full LC and MS methodology can be extracted from the appropriate raw file using XCalibur FreeStyle software or upon request.

Glycopeptide fragmentation data were extracted from the raw file using Byos (Version 4.0; Protein Metrics Inc.). The glycopeptide fragmentation data were evaluated manually for each glycopeptide; the peptide was scored as true-positive when the correct b and y fragment ions were observed along with oxonium ions corresponding to the glycan identified. The MS data was searched using the Protein Metrics 305 N-glycan library with sulfated glycans added manually. The relative amounts of each glycan at each site as well as the unoccupied proportion were determined by comparing the extracted chromatographic areas for different glycotypes with an identical peptide sequence. All charge states for a single glycopeptide were summed. The precursor mass tolerance was set at 4 ppm and 10 ppm for fragments. A 1% false discovery rate (FDR) was applied. The relative amounts of each glycan at each site as well as the unoccupied proportion were determined by comparing the extracted ion chromatographic areas for different glycopeptides with an identical peptide sequence. Glycans were categorized according to the composition detected.

#### GPC-Fab complex formation

Purified GPC-I53-50A was incubated with purified Fabs for at least 1 h at 4°C at a 1:9 molar ratio of GPC-I53-50A to Fab. Next, complexes were purified from unbound Fab by size exclusion chromatography using a Superdex 200 increase 10/300 GL column. Fractions corresponding to GPC-Fab complexes (9-10.5 mL) were pooled and concentrated using a MWCO concentrator with a cutoff of 100 kDa (Millipore).

#### CryoEM grid preparation and imaging

To prepare grids for sample application, UltrAuFoil R1.2/1.3 (Au, 300-mesh; Quantifoil Micro Tools GmbH) grids were treated with Ar/O^2^ plasma using a Solarus plasma cleaner (Gatan) for 10 s or were plasma discharged for 25 s at 15 mA using a PELCO easiGlow (Ted Pella Inc.). Right before applying the protein samples to the grids, we added flouro-octyl maltoside at a final concentration of 0.02% (w/v). Cryo-grids were prepared using a Vitrobot mark IV (Thermo Fisher Scientific). In all instances, the chamber temperature and humidity were set to 4°C and 100%, respectively. Samples were frozen using variable blot times between 3 to 7 s with a blot force of 1 s and a wait time of 10 s. After blotting, the grids were plunge-frozen in liquid ethane.

Cryo-grids were loaded into an FEI Titan Krios or Talos Arctica (Thermo Scientific), which operate at 300 or 200 kV, respectively. Both microscopes were equipped with a K2 Summit direct electron detector camera (Gatan). The data were collected with approximate cumulative exposure of 50 e^-^/Å^2^. Magnifications were set to 130,000 or 36,000X for the Krios and Arctica, respectively. Automated data collection using the Leginon software package^77^ was employed for all datasets reported. Additional information can be found in Table S1.

#### CryoEM data processing

Image preprocessing was performed using the Appion software package.^86^ Micrograph movie frames were first aligned and dose-weighted using the UCSF MotionCor2 software.^78^ Initial data processing was performed in cryoSPARC v3.0^76^ including particle picking and early 2D classification. Quality initial 2D classes were used to inform template picking of the datasets followed by iterative rounds of 2D classification where bad particle picks were removed.

All datasets were analyzed using an initial model generated in UCSF Chimera^74^ from known structures of the LIV GPC (PDB 7SGD) and I53-50A protein (PDB 6P6F). For GPC-I53-50A and Fab complexes, the ligand-free initial model was used for initial 3D refinement steps. After preliminary 3D maps were generated demonstrating Fab density, they were lowpass filtered and used as the initial model for subsequent steps.

For ligand-free GPC-I53-50As, preliminary 3D refinements were performed in cryoSPARC v3.0.^76^ Heterogeneous refinements were used to sort out remaining bad particles and homogenous refinements to orient the GPC appropriately by including the I53-50A scaffold density. Iterative rounds of local refinements were performed with masks that excluded the scaffold density. These particle stacks were transferred to Relion 3.1^81^ for further processing. Local 3D refinements and 3D classifications without global alignment were performed to further polish the particle stack. C3 symmetry was then applied during local 3D refinement followed by CTF refinements. Particle stacks were imported back to cryoSPARC v3.0 for final rounds of C3 local refinement, global CTF refinement, and the final C3 local refinement job. See Figure S3B for more detail.

For Ab-bound GPC-I53-50A structures, the same general processing steps were followed as above sans moving particles to Relion 3.1. LIV GPC-I53-50As bound to 12.1F and S370.7 were analyzed by imposing C3 symmetry after initial alignments. LIV GPC-I53-50A bound to 19.7E was analyzed by symmetry expanding the particle set after C1 alignment along the C3 axis of symmetry. Particles were sorted using focused classification using a 60 Å sphere mask around the epitope-paratope interface to distinguish particles with Fab density. Subsequent refinements were performed to constrain particle alignment to one protomer face.

#### Atomic model building and refinement

Post-processed maps were used to build all final atomic models. For LIV GPC-I53-50As, PDB 7SGD was used as the initial model and manually fit into density using Coot.^92^ Initial models for LII, LV, and LVII GPC-I53-50As were generated using SwissModeler^93^ and manually fit into density using Coot. 12.1F, 19.7E, and S370.7 Fab initial models were produced by ABodyBuilder^82^ and manually fit into the post-processed maps using Coot.^94^ Iterative manual modeling building in Coot followed by Rosetta relaxed refinement were used to generate the final models.^95^ The model fit to map for all models was validated using MolProbity and EMRinger analyses^96^^,^^85^ in the Phenix software package.^84^ Glycan conformation was assessed using Privateer^89^ iteratively until probable orientations were achieved. Epitope-paratope interactions were analyzed using the web-based Epitope-Analyzer.^87^ Glycan involvement at the epitope-paratope was identified structurally through their proximity (<4 Å) to Fabs via UCSF ChimeraX’s clash tool.^75^ Cα RMSD values of GPCs were determined by fitting one GPC protomer per structure (GP1 and GP2 subunit). Cα RMSD values of all residues pairs in the sequence-aligned GP1 models of 12.1F-bound GPC structures (PDBs 8EJH and 7UOV ^44^).The final RMSD values were reported for pruned and unpruned atom pairs in sequence-aligned GP1 and GP2 subunits, excluding glycans. All RMSD values were calculated in ChimeraX using the MatchMaker iterative alignment tool and implementing the Needleman-Wunsch alignment algorithm and BLOSUM-62 similarity matrix. Buried surface area calculations for the fusion peptide were performed using UCSF Chimera.^74^ Buried surface area calculations for Ab interactions were calculated using PDBePISA.^88^ Final atomic models have been submitted to the Protein Data Bank (PDB) with accession codes found in Table S1. All figures featuring atomic models were generated using UCSF ChimeraX.^75^

#### Antibody digestion and Fab purification

Fabs of 19.7E were generated by papain digestion of purified IgG. First, a buffered aqueous suspension of papaya latex papain (Sigma Aldrich) was activated by incubating in 100 mM Tris, 2 mM EDTA, 10 mM L-cysteine at 37°C for 15 mins. Next, IgG was incubated with activated papain in 100 mM Tris, 2 mM EDTA, 10 mM L-cysteine at a ratio of 40 μg activated papain per 1 mg of purified IgG for 5 hours at 37°C. The reaction was quenched by adding iodoacetamide to a final concentration of 0.03 M. Undigested IgG and Fc fragments were removed by a 2 h incubation with CaptureSelect IgG-Fc resin (Thermo Fisher Scientific). Resin was spun down and the supernatant run on a Superdex 200 increase 10/300 GL column (Sigma-Aldrich) size exclusion column using TBS as its running buffer. Fractions from 15.5-16.5 mL elution volume were collected and concentrated in a MWCO concentrator (Millipore) with a 10 kDa cutoff.

#### Antibody affinity measurements using BLI

Ab binding to GPC-I53-50As was assessed using an Octet Red96 instrument (ForteBio). Biotinylated GPC-I53-50A was loaded onto SA sensors (Sartorius) at 100 nM. After a short dip in running buffer (PBS, 0.1% BSA, 0.02% Tween20, pH 7.4), sensors were dipped in IgGs diluted to 400, 200, 100, 50, 25, or 12.5 nM. For Fab measurements, the sensors were dipped in a 400 nM dilution of Fabs. Association and dissociation steps were measured for 600 s. Assays were performed at 30°C. All dilutions were made in running buffer with a final volume of 200 μL per well. Kinetics buffer references were subtracted from all data, which was then analyzed using Octet Data Analysis software. 12.1F, 19.7E, and S370.7 IgG kinetics were modeled assuming a 1:1 binding model while 37.7H assumed a 2:1 binding model because of its biphasic on-rate and poor fit with a 1:1 model.

#### LAMP-1 competition assessment using BLI

Biotinylated GPC-I53-50As diluted in running buffer (PBS, 0.02% Tween20, 0.1% BSA) were loaded onto SA sensors (Sartorius) to a signal of 1.0 nm using an Octet Red96 system (ForteBio). After a short dip in running buffer, the sensors were dipped in 400 nM of 12.1F, 19.7E, S370.7, or 25.10C diluted in running buffer or running buffer alone. To measure IgG dissociation, the sensor was dipped for 1200 s in pH 5.0 running buffer (50 mM NaCitrate, 150 mM NaCl, pH 5.0, 0.1% BSA, 0.02% Tween20). The sensor was then dipped for 600 s in 200 μg/mL of recombinant LAMP-1 ectodomain in pH 5.0 running buffer, after which the sensor was dipped in pH 5.0 running buffer for 1200 s to measure LAMP-1 dissociation.

#### Pseudovirus neutralization assay

LASV pseudoviruses were made as previously described^19^^,^^51^ and pseudovirus neutralization assays were also performed as previously described using LASV psuedotyped viruses and TZM-bI cells.^51^ IC_50_ values were determined as the concentration at which infectivity was inhibited by 50% using Prism 9 (GraphPad).

#### Neutralization assay using authentic LASV

Neutralization assays using authentic LASV (lineage IV, strain Josiah) were performed in the BSL-4 laboratory of the Institute of Virology, Philipps University Marburg, Germany as previously described.^51^

#### Antibody quaternary preference assay using BLI

12.1F, 19.7E, 37.7H, and S370.7 IgGs were immobilized on AHC sensors (Sartorius) to a signal of 1.0 nM using an Octet Red96 instrument (ForteBio). The immobolized IgGs were then dipped in running buffer (PBS, 0.1% BSA, 0.02% Tween20, pH 7.4) followed by LIV GPC-I53-50A trimer, LIV GPC monomer, or running buffer. LIV GPC-I53-50A trimer and LIV GPC monomer were diluted in running buffer to concentrations that would contain the same amount of protomers in solution: 150 nM and 450 nM, respectively. Following a 600 s association period, the tips were dipped into running buffer and dissociation was measured for 600 s.

#### Synthetic matriglycan microarray printing and screening

The synthesis of matriglycan compounds were reported previously.^36^ All compounds were printed on NHS-ester activated glass slides (NEXTERION® Slide H, Schott Inc.) using a Scienion sciFLEXARRAYER S3 non-contact microarray equipped with a Scienion PDC80 nozzle (Scienion Inc.). Individual compounds were dissolved in sodium phosphate buffer (0.225 M, pH 8.5) at the desired concentration and were printed in replicates of 6 with spot volume ∼ 400 pL, at 20°C and 50% humidity. Each slide has 24 subarrays in a 3x8 layout. After printing, slides were incubated in a humidity chamber for 8 hours and then blocked for one hour with a 5 mM ethanolamine in a Tris buffer (pH 9.0, 50 mM) at 50°C. Blocked slides were rinsed with DI water, spun dry, and kept in a desiccator at room temperature for future use.

Printed glass slide was pre-blocked with a solution of 1x TSM binding buffer (20 mM Tris·HCl, pH 7.4, 150 mM NaCl, 2 mM CaCl2, and 2 mM MgCl2, 0.05% Tween-20, 1% BSA) for 90 mins and the blocking solution was discarded. The Strep-tagged GPC-I53-50A featuring the native S1P cleavage site (1 μg/mL) was incubated with 12.1F, 19.7E, 25.10C, 37.7H, or S370.7 mAbs (5 μg/mL) in TSM binding buffer at 4°C for 1 h before StrepMAB-Classic Oyster 645 conjugate (0.5 μg/mL, IBA Lifesciences 2-1555-050) was added, and the solution was further incubated for another 30 min at 4°C. For the detection of the mAb, a Cy3 conjugated goat-anti-human IgG Ab was used (5 μg/mL, Jackson Immuno Research, 109-165-008). The solution was then added to the microarray slide and the slide was incubated at room temperature for 1 h. The slide was sequentially washed with TSM wash buffer (20 mM Tris·HCl, pH 7.4, 150 mM NaCl, 2 mM CaCl2, and 2 mM MgCl2, 0.05% Tween-20), TSM buffer (20 mM Tris·HCl, pH 7.4, 150 mM NaCl, 2 mM CaCl2, and 2 mM MgCl2) and water.

The slides were scanned using a GenePix 4000B microarray scanner (Molecular Devices) at the appropriate excitation wavelength with a resolution of 5 μM. Various gains and PMT values were employed in the scanning to ensure all the signals were within the linear range of the scanner’s detector and there was no saturation of signals. The image was analyzed using GenePix Pro 7 software (version 7.2.29.2, Molecular Devices). The data was analyzed with an Excel macro (https://doi.org/10.5281/zenodo.5146251) to provide the results. The highest and lowest value of the total fluorescence intensity of the six replicates spots were removed, and the four values in the middle were used to provide the mean value and standard deviation. Due to the small sample size normality of this data was not assumed. Instead, two-tailed Mann-Whitney *U*-tests were performed to compare matriglycan binding to GPC-I53-50A in different conditions. *P* value estimations using this test are capped at 0.029 based on the sample size.

#### B-cell sorting

We used two GPC bait constructs for isolating LASV-specific B cells: LIV GPC-I53-50A and Josiah rGPe^19^ with a T4-foldon domain. Biotinylated antigens were barcoded by incubation with barcoding complexes (TotalSeq-C, BioLegend) at a 2:1 molar ratio, resulting in an average of 2 antigen molecules per antigen-barcode complex (AgBC). We separately produced two AgBCs for each antigen using different fluorophores (APC and PE) and different barcodes to allow more stringent FACS selection and downstream data analysis. Previously cryopreserved PBMCs from a Sierra Leonean survivor of Lassa Fever (donor 1102370) were first stained with a “dark” human serum albumin AgBC (containing a barcode oligo but no fluorophore) prior to labeling with barcoded antigen baits and a small panel of flow cytometry Abs (anti-CD19 and a dump channel containing anti-CD3 and anti-CD14). All B cells (CD19+CD3-CD14-) double-positive for APC and PE were bulk sorted using a FACSMelody cell sorter (Beckton Dickinson). Antigen-selected B cells were then immediately processed on a 10x Genomics Chromium Controller using Next GEM 5’ v2 reagents as previously described.^97^ The resulting single cell sequencing libraries (gene expression, feature barcode and VDJ-B) were sequenced on an Illumina NovaSeq 6000 using a 100-cycle SP v1.5 reagent kit. Raw sequencing data was processed with CellRanger^79^ and Ab sequences were annotated using the ab[x] toolkit.^98^ Specificity classification was determined from AgBC data using scab.^97^

### Quantification and statistical analysis

The number of replicates (*n*), definition of center, dispersion measures (when applicable), and the statistical tests employed are noted in figure legends and/or results. Notably, for the synthetic matriglycan arrays, the small sample size insinuates the normality of the data cannot be assumed. Two-tailed Mann-Whitney *U*-tests were thus performed. *P* value estimations using this test are capped at 0.029 based on the sample size, and we consider *P* values <0.05 to be significant. All statistical analyses were performed using Graphpad Prism 8.0.

## Data Availability

• Maps generated from the electron microscopy data are deposited in the Electron Microscopy Databank (http://www.emdatabank.org/) under accession IDs EMD-28178, EMD-28170, EMD-28180, EMD-28181, EMD-28182, EMD-28183, and EMD-28184. Atomic models corresponding to these maps have been deposited in the Protein DataBank (http://www.rcsb.org/) under accession IDs 8EJD, 8EJE, 8EJF, 8EJG, 8EJH, 8EJI, and 8EJJ. Mass spectrometry raw files have been deposited in the MassIVE proteomics619database and can be accessed through accession number: MSV000091003. Nucleotide sequences of S370.7 heavy and lambda chains can be accessed via GenBank: OQ451467 and OQ451468, respectively. The raw data reported in this study will be shared by the corresponding author upon request. • This paper does not report original code. • Any additional information required to reanalyze the data reported in this work paper is available from the lead contact upon request.

## Supplemental information

Document S1. Figures S1–S11 and Tables S1–S5

Document S2. Article plus supplemental information
