# Supplementary material for: Structural conservation of Lassa virus glycoproteins and recognition by neutralizing antibodies
Source: Cell Rep. 2023 May 18;42(5):112524. doi: 10.1016/j.celrep.2023.112524 (PMC10242449; doi:10.1016/j.celrep.2023.112524)
Supplement: Document S1. Figures S1–S11 and Tables S1–S5 [file mmc1.pdf]

Cell Reports, Volume *42*

Supplemental information

Structural conservation of Lassa virus glycoproteins and recognition by neutralizing antibodies

Hailee R. Perrett, Philip J.M. Brouwer, Jonathan Hurtado, Maddy L. Newby, Lin Liu, Helena Müller-Kräuter, Sarah Müller Aguirre, Judith A. Burger, Joey H. Bouhuijs, Grace Gibson, Terrence Messmer, John S. Schieffelin, Aleksandar Antanasijevic, Geert-Jan Boons, Thomas Strecker, Max Crispin, Rogier W. Sanders, Bryan Briney, and Andrew B. Ward

Supplemental Information

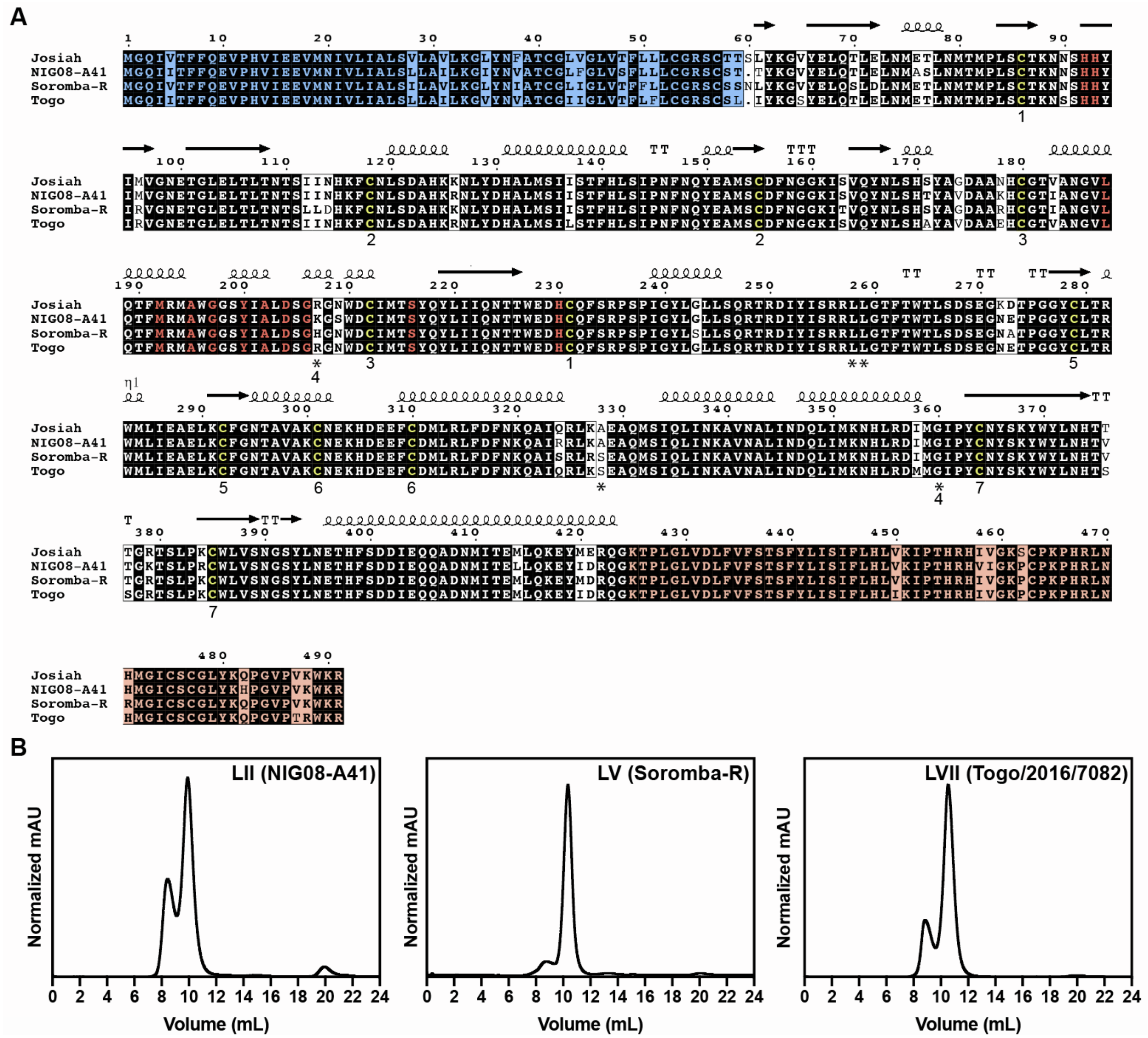

Fig. S1: GPC lineage sequences and purification as trimers. Related to Figure 1. (A) Native amino acid sequences from the four GPCs investigated in this study (LIV, Josiah; LII, NIG08-A41, LV, Soromba-R, and LVII, Togo) were aligned using the T-Coffee multiple sequence alignment server.[1] Residues shown as white text on a black background are conserved between the four strains. The stable signal peptide (SSP) is highlighted in blue and the transmembrane domain in orange. The histidine triad and additional residues required for LAMP-1 binding are highlighted in red.[2,3] Ectodomain residues involved in disulfide bonds are highlighted yellow and numbered below. GPCysR4 stabilizing mutation[4] locations are indicated with asterisks below. Secondary structural features are depicted above residues based on the atomic model for LIV GPC (PDB 8EJD). Accession codes for the sequences are as follows: Josiah, NP_694870.1; NIG08-A41, ADU56626.1; Soromba-R, AHC95553.1; and Togo/2016/7082, AMR44577.1. Sequence alignment visualized using ESPript3.0[5]. (B) SEC chromatograms of LII, LV, and LVII GPC-I53-50As.

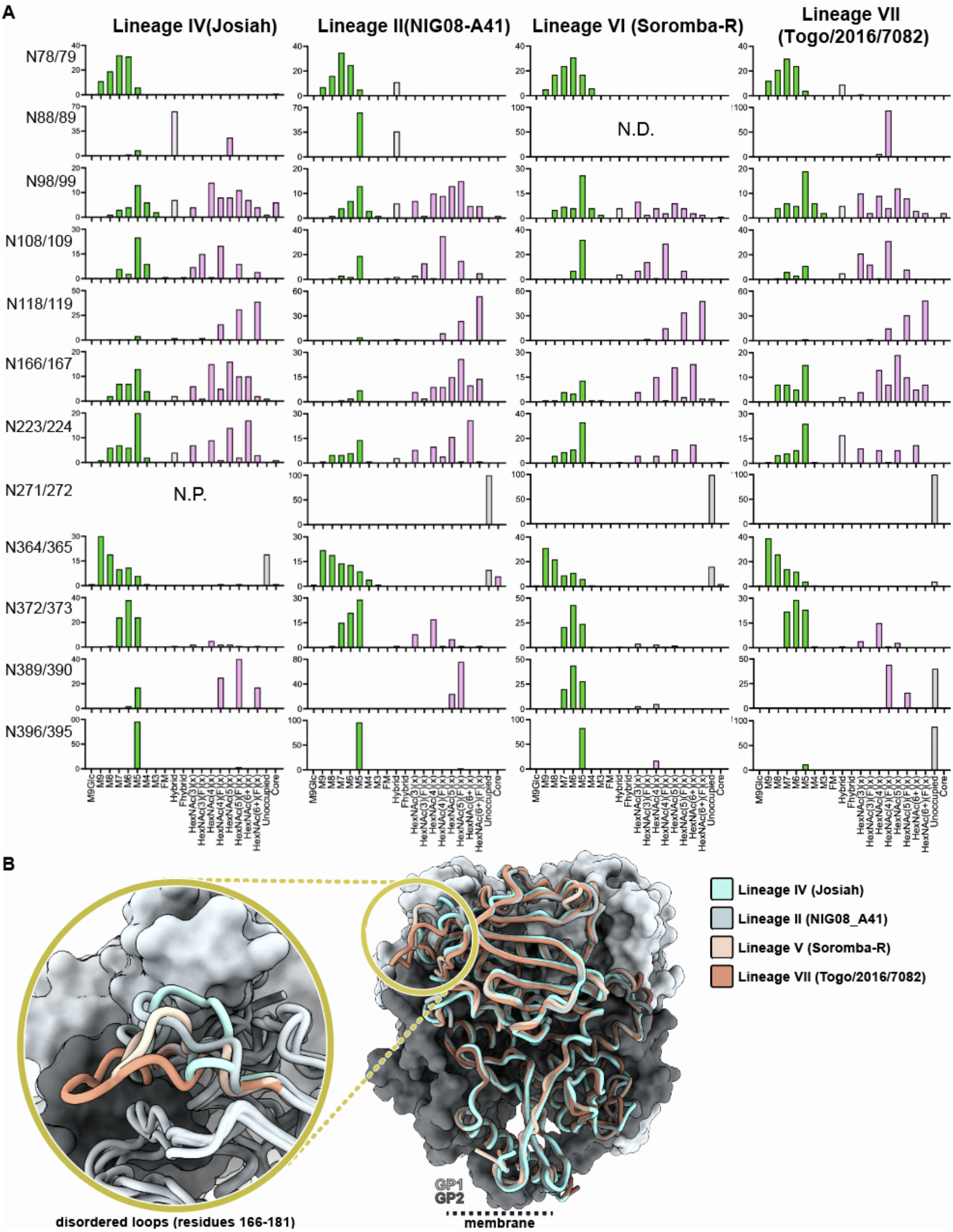
A
Lineage IV(Josiah)
Lineage II(NIG08-A41)
Lineage VI (Soromba-R)
Lineage VII (Togo/2016/7082)
N78/79
N88/89
N98/99
N108/109
N118/119
N166/167
N223/224
N271/272
N364/365
N372/373
N389/390
N396/395
N.D.
N.P.
M9Glc
M9
M8
M7
M6
M5
M4
M3
FM
Hybrid
Fhybrid
HexNAc(3)(x)
HexNAc(3)(F)(x)
HexNAc(4)(x)
HexNAc(4)(F)(x)
HexNAc(5)(x)
HexNAc(5)(F)(x)
HexNAc(6+)(x)
HexNAc(6+)(F)(x)
Unoccupied
Core
B
Lineage IV (Josiah)
Lineage II (NIG08_A41)
Lineage V (Soromba-R)
Lineage VII (Togo/2016/7082)
GP1
GP2
membrane
disordered loops (residues 166-181)

Fig. S2: Detailed glycan analyses and further GPC structure visualization. Related to Figure 2. (A) Quantification and identities of glycan types determined by LCMS for each PNGS of the LASV lineages. Oligomannose-type glycans are shown in green, hybrid in dashed pink, complex glycans in pink and unoccupied sites in gray. N.D. indicates a PNGS which was undetected in the assay. N.P. indicates no PNGS is present in the sequence at that site. HexNAc(2)Hex(9−5) was classified as M9 to M3. Any of these structures containing a fucose were categorized as FM (fucosylated mannose). HexNAc(3)Hex(5−6)X was classified as Hybrid with HexNAc(3)Hex(5-6)Fuc(1)X classified as Fhybrid. Complex-type glycans were classified according to the number of HexNAc subunits and the presence or absence of fucosylation. As this fragmentation method does not provide linkage information, compositional isomers are grouped. For example, a triantennary glycan contains $HexNAc_5$ but so does a biantennary glycans with a bisect. Core glycans refer to truncated structures smaller than M3. M9Glc-M4 were classified as oligomannose-type glycans. (B) Comparison of the disordered loops (LIV and LV residues 166-181; LII and LVII residues 165-180).

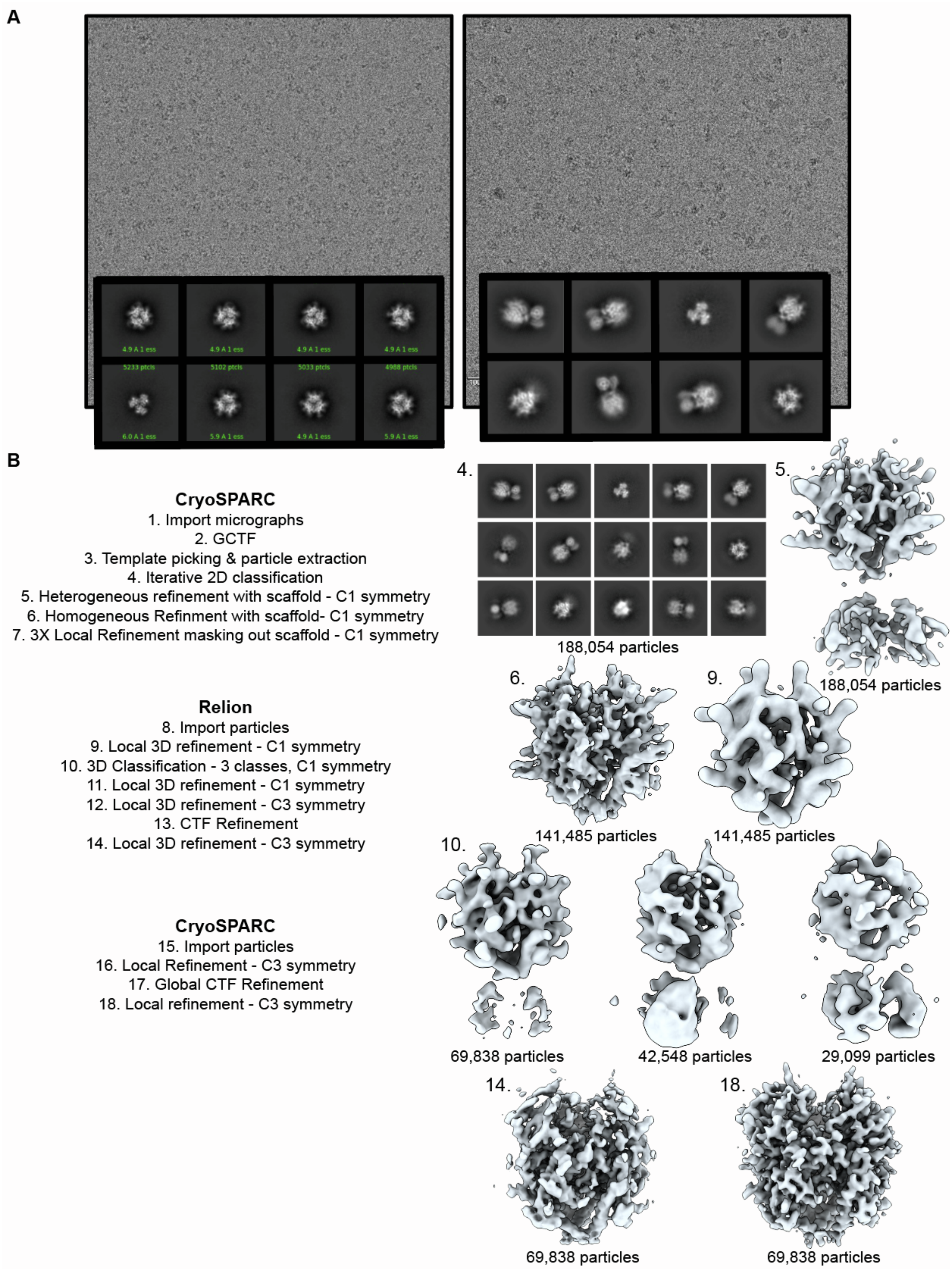
A
B
CryoSPARC
1. Import micrographs
2. GCTF
3. Template picking & particle extraction
4. Iterative 2D classification
5. Heterogeneous refinement with scaffold - C1 symmetry
6. Homogeneous Refinment with scaffold- C1 symmetry
7. 3X Local Refinement masking out scaffold - C1 symmetry
Relion
8. Import particles
9. Local 3D refinement - C1 symmetry
10. 3D Classification - 3 classes, C1 symmetry
11. Local 3D refinement - C1 symmetry
12. Local 3D refinement - C3 symmetry
13. CTF Refinement
14. Local 3D refinement - C3 symmetry
CryoSPARC
15. Import particles
16. Local Refinement - C3 symmetry
17. Global CTF Refinement
18. Local refinement - C3 symmetry
4.
188,054 particles
5.
188,054 particles
6.
141,485 particles
9.
141,485 particles
10.
69,838 particles
42,548 particles
29,099 particles
14.
69,838 particles
18.
69,838 particles

Fig. S3: Optimizing single-particle cryoEM for GPC samples. Related to Figure 2. (A) Sample micrographs showing effectiveness of fluoryl-octyl maltoside in improving GPC orientation in vitreous ice (right) compared to other detergents such as LMNG (left). (B) Representative data processing overview schematic showing data from EMD-28179. A similar processing scheme was employed for EMD- 28178, EMD-28180, and EMD-28181.

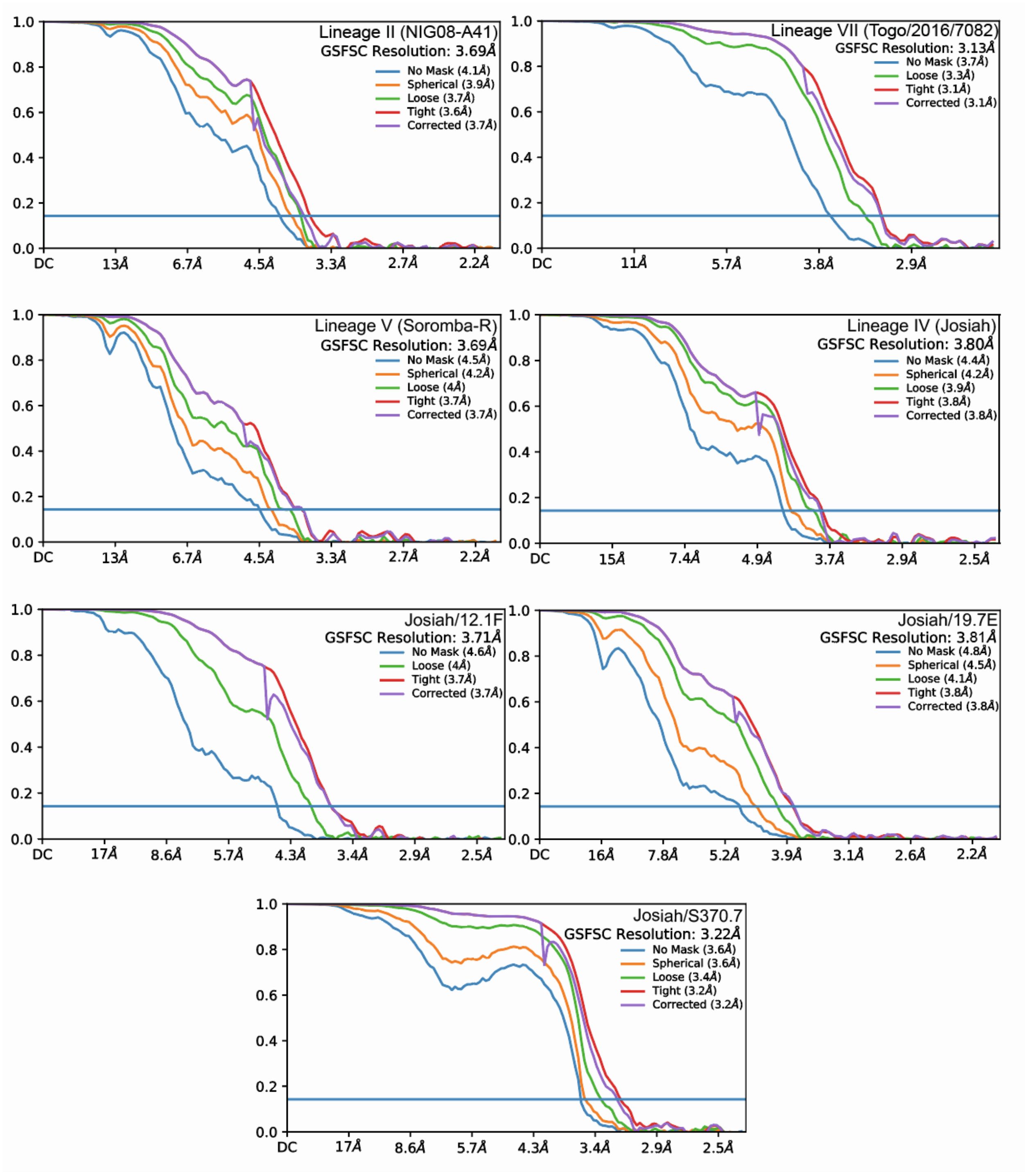

Fig. S4: FSC plots for EM maps of LASV GPCs and GPCs bound to 12.1F, 19.7E, and S370.7. Related to Figures 2, 4, and 6. Reported resolutions coincide with an FSC cutoff of 0.143. Plots were generated in cryoSPARC 3.2.[6]

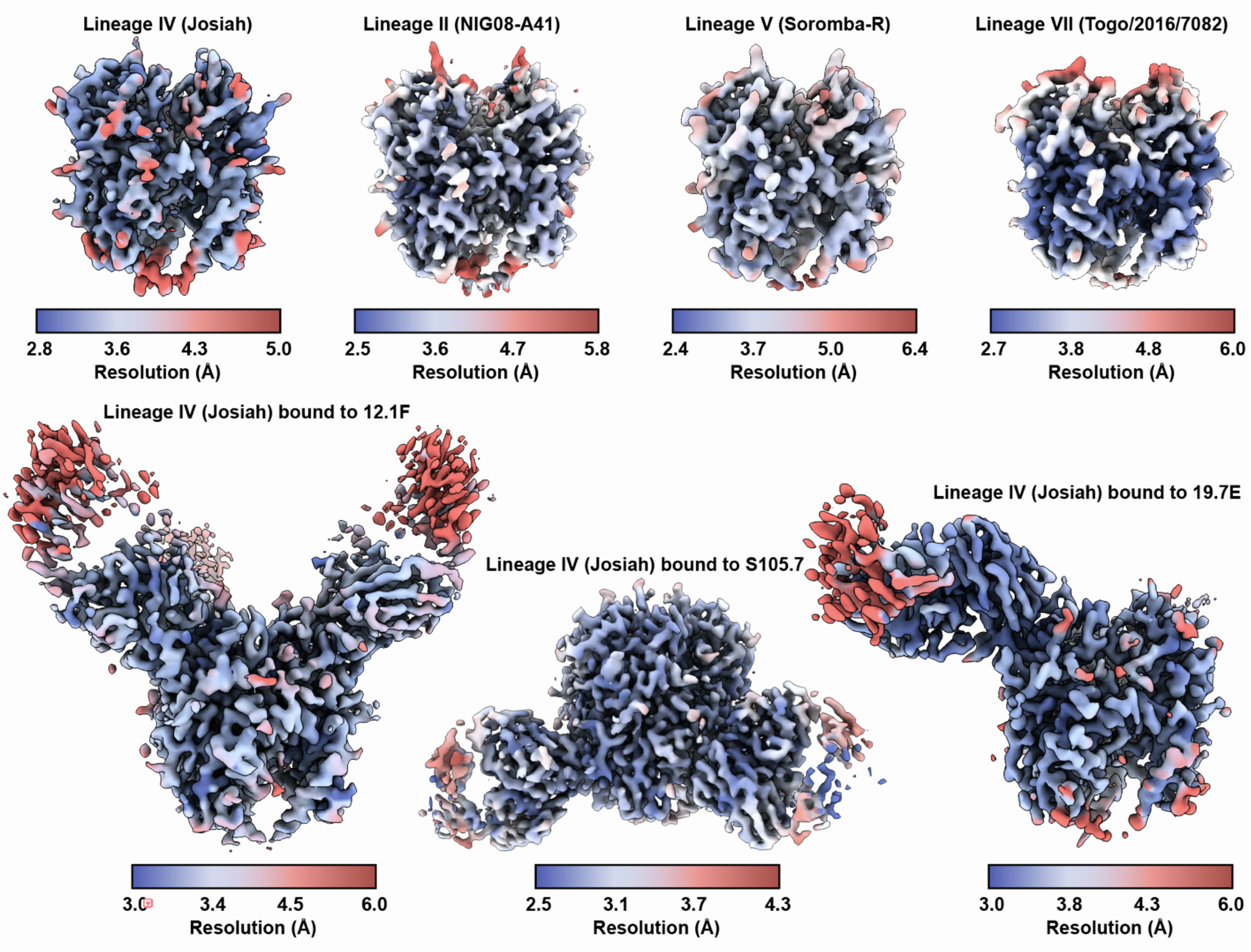

Fig. S5: Local resolution plots for EM maps of LASV GPCs and GPCs bound to 12.1F, 19.7E, and S370.7. Related to Figures 2, 4, and 6. Local resolution was calculated according to a 0.143 FSC threshold in cryoSPARC 3.2 [6] and visualized in ChimeraX.[7]

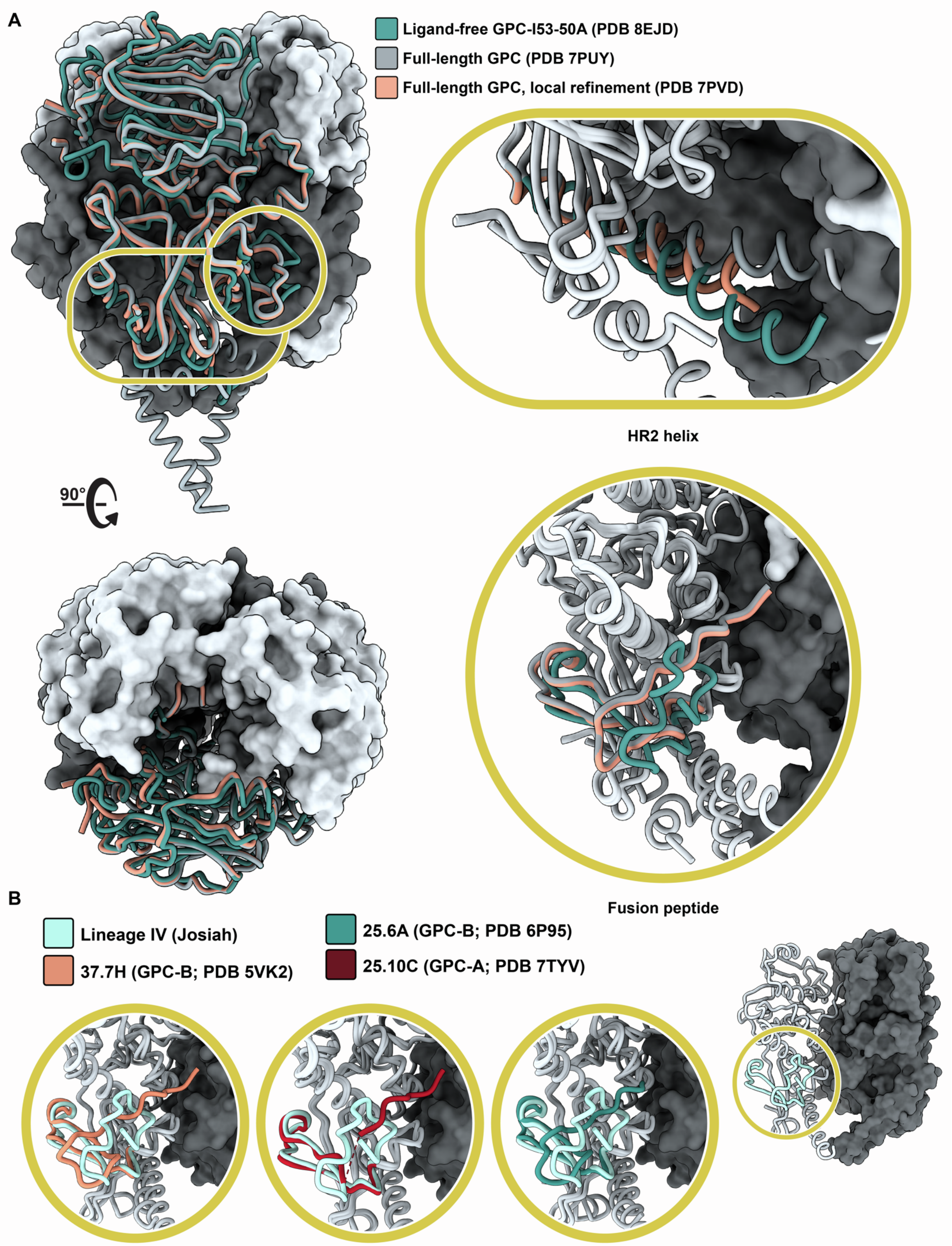
A
Ligand-free GPC-I53-50A (PDB 8EJD)
Full-length GPC (PDB 7PUY)
Full-length GPC, local refinement (PDB 7PVD)
HR2 helix
90°
Fusion peptide
B
Lineage IV (Josiah)
37.7H (GPC-B; PDB 5VK2)
25.6A (GPC-B; PDB 6P95)
25.10C (GPC-A; PDB 7TYV)

Fig. S6: Comparison of GPC-I53-50A to full-length GPC and fusion peptide analysis. Related to Figure 2. (A) Ligand-free LIV GPC-I53-50A (PDB 8EJD) compared to full-length, native GPC (PDBs 7PUY and 7PVD) bound to primary host cell receptor matriglycan (top left).[8] The apex of the GPC trimer is shown at the bottom left, where the native fusion site is modeled. Structural differences in the HR2 helix (top right) and fusion peptide (bottom right) are shown. (B) Ligand-free LIV GPC (PDB 8EJD) overlaid with additional GPC-B-specific Abs 37.7H and 25.6A and GPC-A-targeting Ab 25.10C.[4,9,11] The putative fusion peptides (residues 260-298) are colored for each model.

A

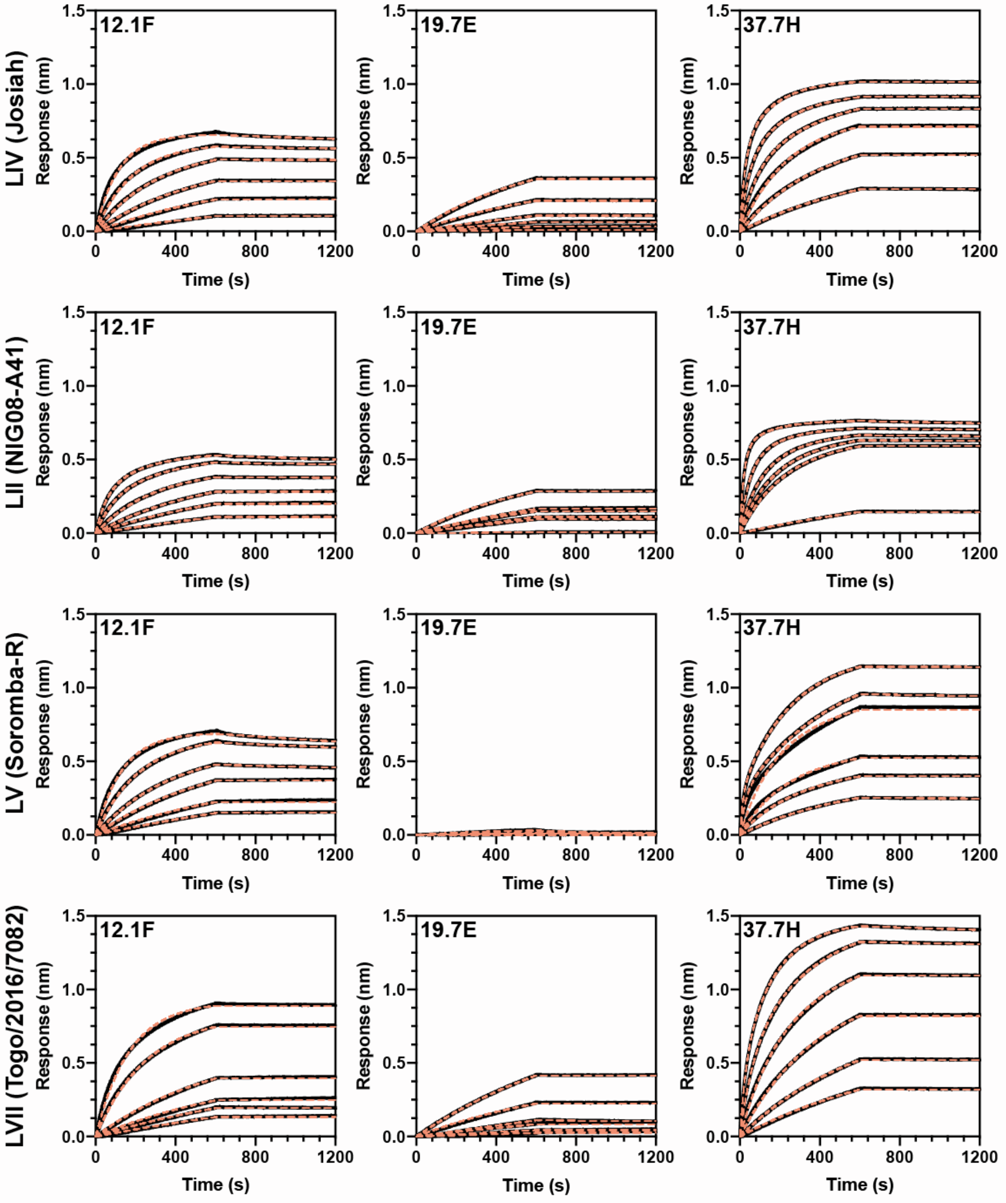

B

k_{on} ($M^{-1}s^{-1}$)

	12.1F	19.7E	37.7H
**Lineage IV (Josiah)**	3.7E+04 ± 4.9E+03	1.5E+04 ± 2.0E+03	6.9E+04 ± 4.2E+03
**Lineage II (NI08-A41)**	2.9E+04 ± 2.8E+03	1.3E+04 ± 2.6E+03	9.9E+04 ± 5.0E+03
**Lineage V (Soromba-R)**	3.3E+04 ± 2.2E+03	N/A	1.4E+05 ± 7.7E+03
**Lineage VII (Togo/2016/7082)**	3.1E+04 ± 2.4E+03	1.3E+04 ± 2.5E+03	4.3E+04 ± 5.7E+03

C

R_{max} (nm)

	12.1F	19.7E	37.7H
**Lineage IV (Josiah)**	0.68	0.37	1.02
**Lineage II (NI08-A41)**	0.53	0.29	0.76
**Lineage V (Soromba-R)**	0.71	0.03	1.15
**Lineage VII (Togo/2016/7082)**	0.91	0.42	1.44

R_{max}:$R_{max,37.7H}$

	12.1F	19.7E	37.7H
**Lineage IV (Josiah)**	0.66	0.36	1.00
**Lineage II (NI08-A41)**	0.52	0.28	1.00
**Lineage V (Soromba-R)**	0.62	0.03*	1.00
**Lineage VII (Togo/2016/7082)**	0.63	0.29	1.00
**Average**	0.61	0.31	1.00

*excluded from average

Fig. S7: Binding profiles of GPC-I53-50A trimers to NAbs 12.1F, 19.7E, and 37.7H. Related to Figure 3. (A) BLI sensorgrams of immobilized biotinylated GPC-I53-50A binding to indicated IgGs at concentrations of 400, 200, 100, 50, 25, and 12.5 nM (black). Dotted orange lines represent the fit used to calculate on-rates and R_{max} values. (B) Kinetics table indicating the k_{on} rates for mAbs per lineage based on data shown in (A). (C) R_{max} values for each mAb at a concentration of 400 nM (left) and the ratios of these R_{max} values to the R_{max} of 37.7H (right). The R_{max}:$R_{max,37.7H}$ ratio was rounded to the nearest third for the predicted binding stoichiometries indicated in Figure 3A.

A

IC50 (nM)	12.1F		19.7E	
	IgG	Fab	IgG	Fab
Lineage IV (Josiah)	0.32	89.5	1.23	>250
Lineage II (NI08-A41)	1.93	36.3	0.68	>250
Lineage III (CSF)	1.40	7.32	0.85	>250
BG505 T332N	>250	>250	>250	>250

More potent — Less potent; Not neutralizing

B

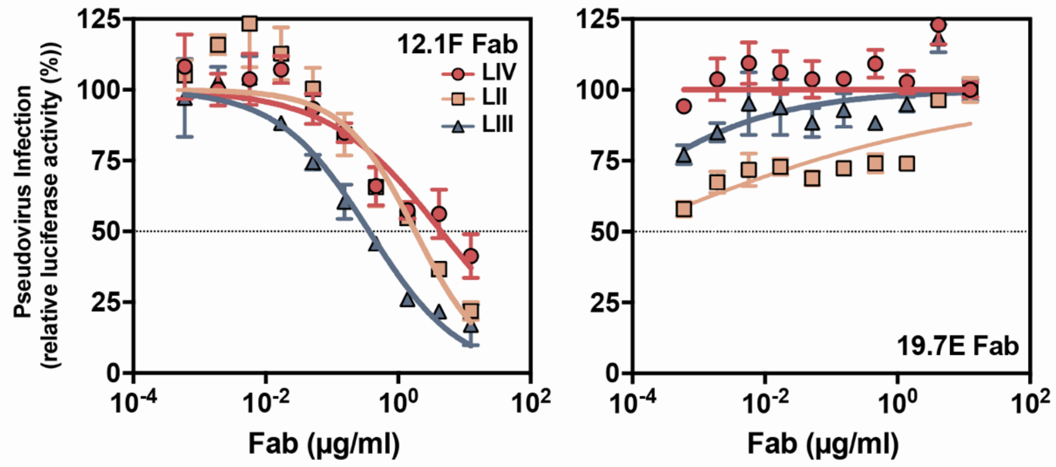

Fig. S8: IC_{50} summary table and Fab neutralization assays. Related to Figure 3. (A) IC50 summary table of pseudovirus neutralization using 12.1F and 19.7E, IgG and Fabs. IC50 values are shown as nM concentrations. (B) Pseudovirus neutralization of LASV lineages by 12.1F and 19.7E Fab. Data points represent the mean with error bars indicating the SEM of three technical replicates.

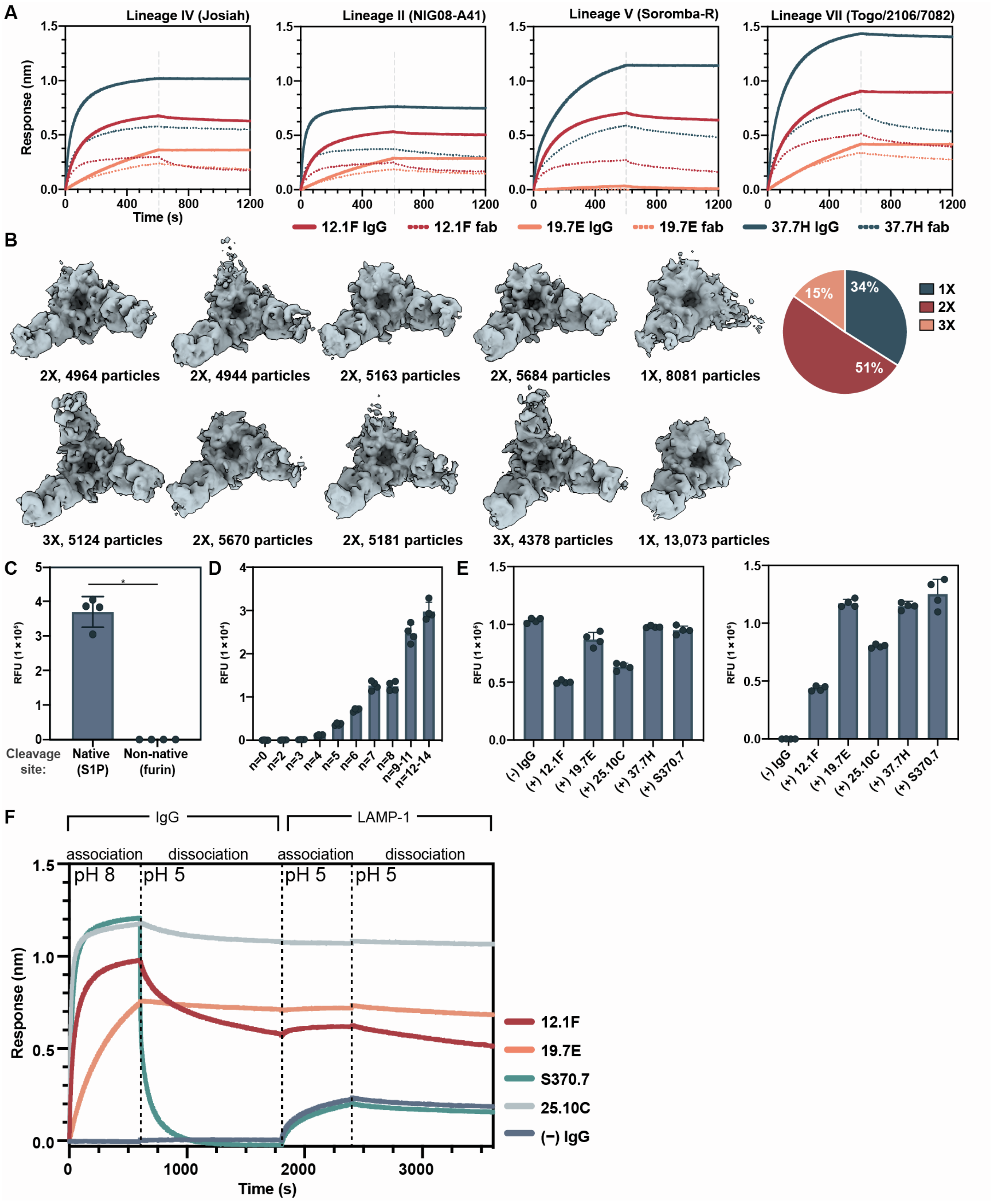

Fig. S9: IgG versus Fab binding, and further details on the matriglycan and LAMP-1 binding assays. Related to Figures 3, 4, and 5. (A) BLI sensorgrams indicating binding behavior of immobilized GPC-I53-50A trimer binding to 400 nM of IgG or Fab. (B) 3D classification of final particle stack used in the 12.1F-bound GPC structure (left). Particles were classified into 10 classes using a ligand-free GPC-I53-50A as the initial model. Because particles were taken from the final

reconstruction, no additional alignment was performed. The pie chart indicates the estimated distribution of GPC particles with 1, 2, or 3 12.1F Fabs bound. (C) Synthetic matriglycan microarray shows preference for GPCs featuring the native S1P cleavage site compared to the engineered furin cleavage site (median RFU of 3.8×10^5 versus 1.9×10^2, respectively; two-tailed Mann-Whitney U-test; $p = 0.029$). (D) GPC-I53-50As with the native S1P cleavage site show length-dependent binding to synthetic matriglycan on the microarray. Here, *n* indicates the number of xylose and glucuronic acid disaccharide repeating units. (E) Synthetic matriglycan assay used to distinguish the effect of bound mAbs on matriglycan binding to GPC-I53-50A with the S1P cleavage site. Bound Strep-tagged trimers were detected using a StrepMab Ab (left) to assess matriglycan binding. The StrepMab Ab is specific for the GPC-I53-50A trimer in this assay as shown by its detection using a Cy3 conjugated goat-anti-human IgG Ab (right). (F) Experimental set-up for BLI LAMP-1 competition assay shown in Fig. 3E and 5F. MAbs 25.10C, 12.1F, and S370.7 show notable dissociation at pH 5. While S370.7 completely dissociates at pH 5, the other mAbs retained some level of binding after 20 minutes at pH 5.

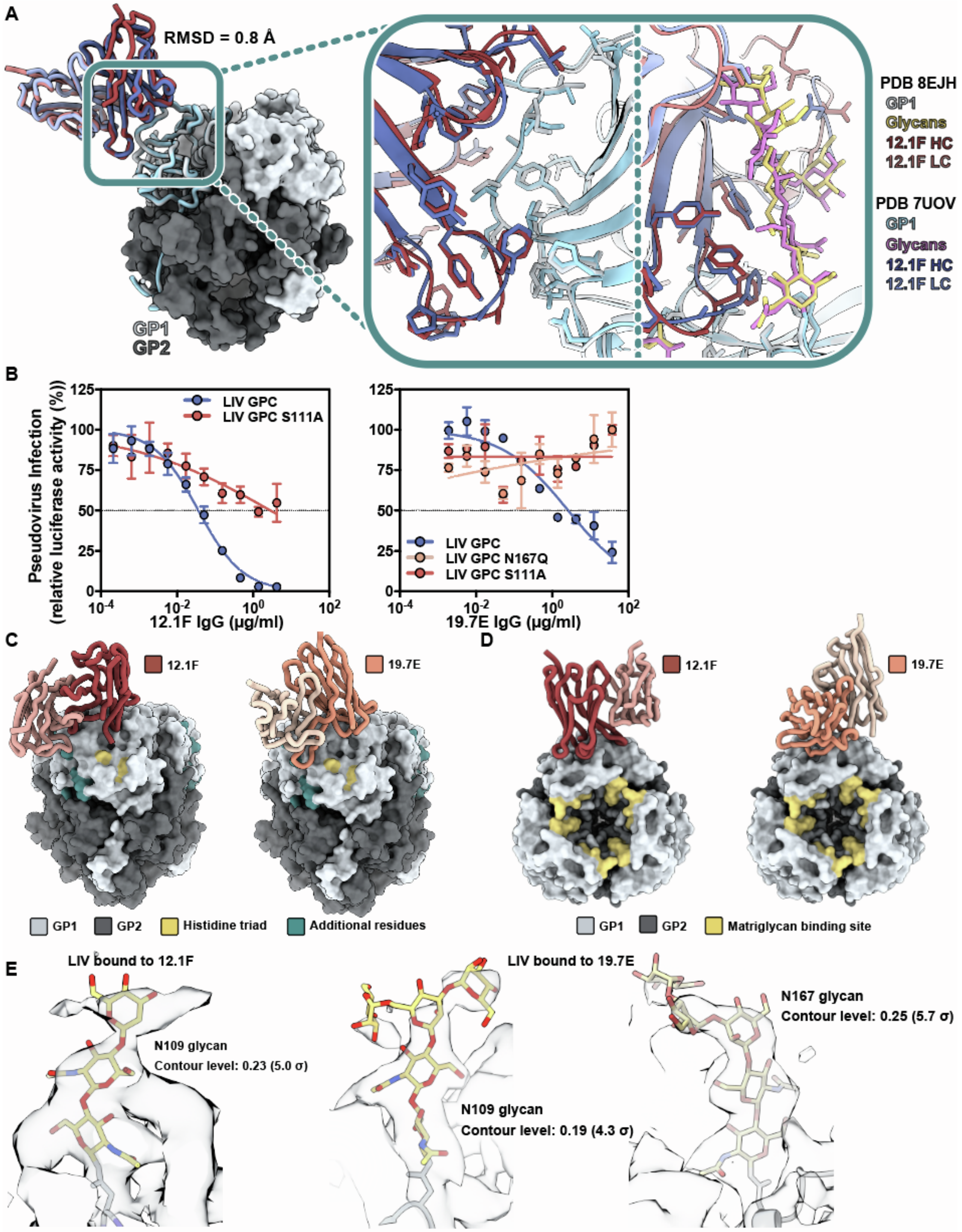

Fig. S10: 12.1F and 19.7E glycan dependence and binding with respect to matriglycan and putative LAMP-1 binding sites. Related to Figure 4. (A) Comparison of 12.1F-bound GPC-I53-50A with recently published 12.1F-and-37.2D-bound GPC.[10] Inset depicts key epitope-paratope interactions of both structures. (B) Pseudovirus neutralization of LASV lineages by indicated mAbs to LIV pseudovirus containing the glycan knockout mutations S111A or N167Q. The dotted line indicates 50% neutralization. Data points represent the mean with error bars indicating the SEM of three (12.1F) or two (19.7E) technical replicates. (C) 12.1F and 19.7E Fabs overlaid on PDB 8EJD with residues important for LAMP-1 binding[2,3] shown

in teal and gold. (D) 12.1F and 19.7E Fabs overlaid on PDB 8EJD with residues important for matriglycan binding shown in gold.[8] (E) Glycan densities in cryo-EM data for glycans required for 12.1F (left) and 19.7E (right) binding.

A

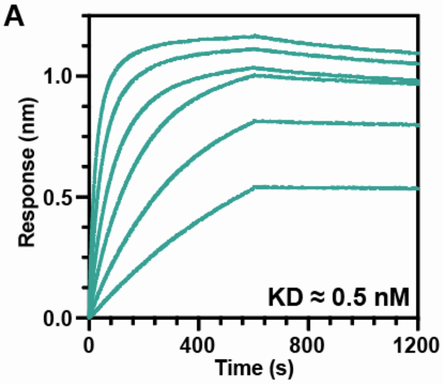

Conc (nM)	k_{on}		KD		R^2
**50**	1.1E+05	± 1.7E+02	3.0E-10	± 4.0E-12	0.990
**25**	1.19+e5	± 1.3E+02	1.3E-10	± 1.3E-12	0.998
**12.5**	1.5E+05	± 1.4E+03	N/A		0.995

C

Replicate	1	2	3	4
Neutralizing titer (µg/mL)	>20	>20	>20	>20

B

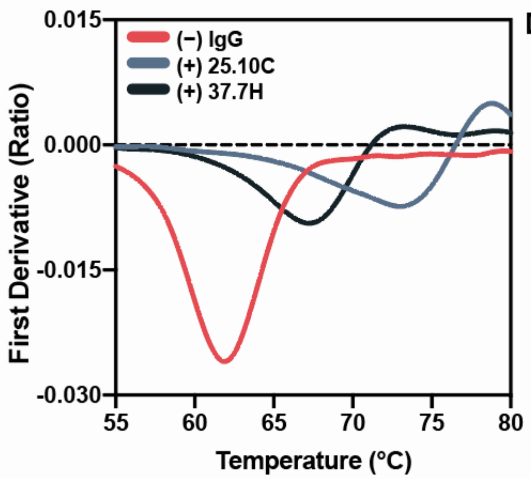

D

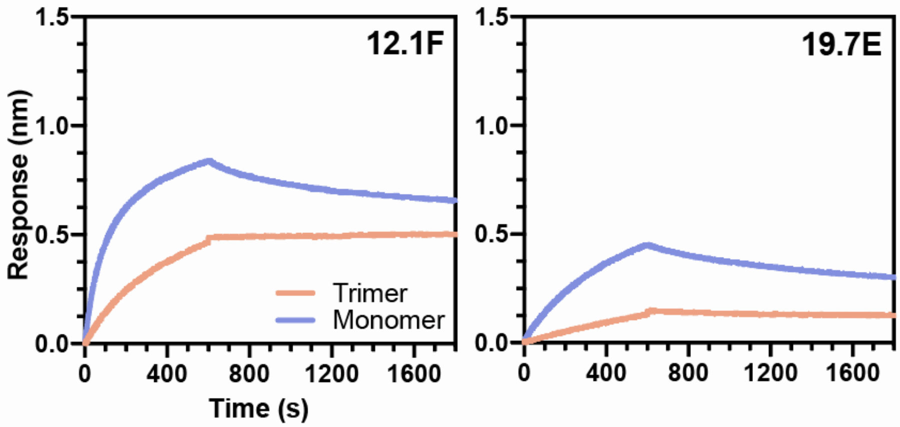

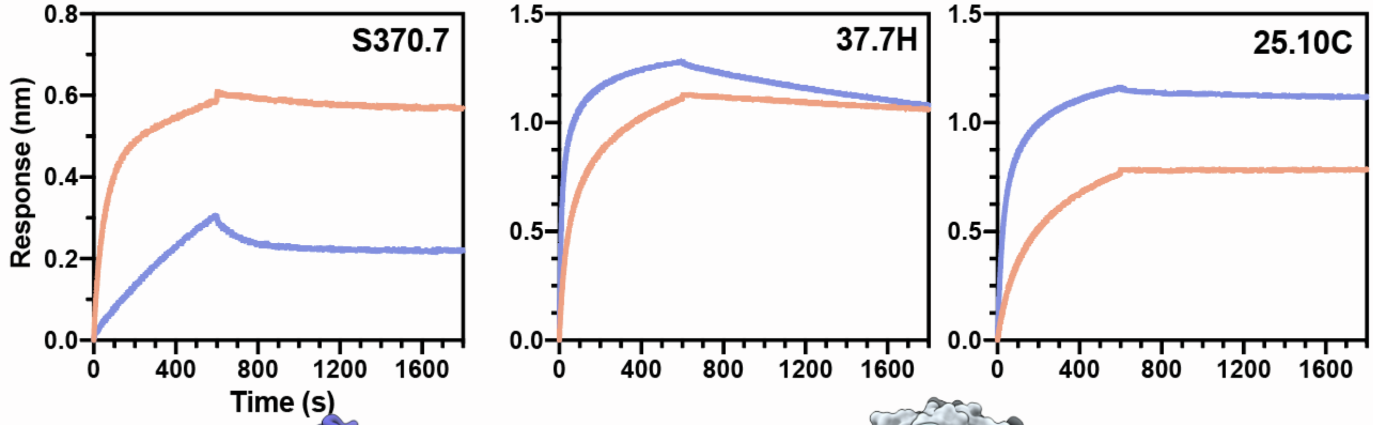

E

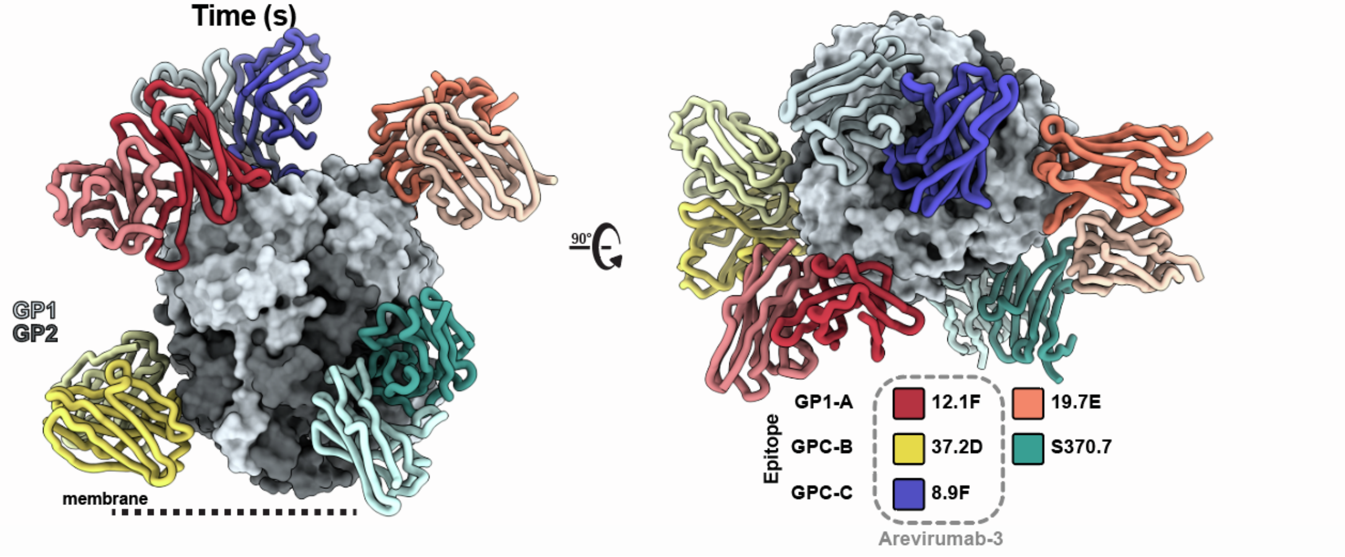

Fig. S11: Biophysical comparison of S370.7 to known nAbs. Related to Figure 6. (A) BLI sensorgrams (left) of immobilized biotinylated LIV GPC-I53-50A binding to S370.7 at concentrations of 400, 200, 100, 50, 25, and 12.5 nM (black). Kinetics table (right) featuring values derived from 1:1 binding model fitting of the raw data that best fit a 1:1 binding model. (B) Thermostability of LIV GPC-I53-50A and LIV GPC-I53-50A in complex with 25.10C or 37.7H Fabs assessed by nanoDSF. Each melting curve is a representative of triplicate curves. (C) Endpoint neutralization titers against authentic, LIV strain Josiah virus. (D) BLI sensorgrams comparing immobilized IgG binding to GPC-I53-50A trimer or GPC monomer. Trimer and monomer were diluted to 150 and 450 nM, respectively, which represents equivalent concentrations of protomer. (E) Comparison of epitopes targeted by S370.7 and 19.7E with those in the Arevirumab-3 cocktail containing 12.1F, 8.9F, and 37.2D (PDBs 7UOT and 7UOV).[10]

Table S1: CryoEM map and atomic model refinement. Related to Fig. 2, 4, and 6.

	Lineage IV (Josiah)	Lineage II (NIG08-A41)	Lineage V (Soromba-R)	Lineage VI (Togo/2016/7082)	Josiah bound to 12.1F fab	Josiah bound to 19.7E fab	Josiah bound to S370.7 fab
**Access codes**							
PDB	8EJD	8EJE	8EJF	8EJG	8EJH	8EJI	8EJJ
EMDB	EMD-28178	EMD-28179	EMD-28180	EMD-28181	EMD-28182	EMD-28183	EMD-28184
Genebank	NP_694870.1	ADU56626.1	AHC95553.1	AMR44577.1	NP_694870.1	NP_694870.1	NP_694870.1
**Data collection and processing**							
Microscope	Talos Arctica	Titan Krios	Titan Krios	Talos Arctica	Talos Arctica	Titan Krios	Talos Arctica
Magnification	36,000	130,000	130,000	36,000	36,000	130,000	36000
Voltage (kV)	200	300	300	200	200	300	200
Electron exposure ($e^-/Å^2$)	50.0	49.2	49.2	50.3	50.3	50.2	50.1
Defocus range (μm)	-0.7 to -2	-0.7 to -2	-0.7 to -2	-0.7 to -2	-0.7 to -2	-0.7 to -2	-0.7 to -2
Pixel size (Å)	1.150	1.045	1.045	1.150	1.150	1.045	1.150
Imposed Symmetry	C3	C3	C3	C3	C3	C1	C3
Final particle number	56,496	69,838	27,663	71,504	62,262	70,071 (symmetry expanded)	96,449
Map resolution (Å)	3.8	3.7	3.7	3.1	3.7	3.8	3.2
FSC Threshold	0.143	0.143	0.143	0.143	0.143	0.143	0.143
Map sharpening B-factor ($Å^2$)	-70	-93	-67	-50	-130	-120	-63
**Model refinement and validation**							
Total Residues	1173	1134	1170	1185	1860	1335	1830
Amino-acids	1086	1068	1080	1083	1761	1257	1743
Carbohydrates	87	66	90	102	99	78	87
RMSD Bonds	0.021	0.022	0.023	0.024	0.020	0.021	0.023
RMSD Angles	1.78	2.11	1.95	1.87	1.75	1.81	1.77
**Ramachandran**							
Outliers (%)	0	0	0	0	0	0	0
Allowed (%)	4.5	6.0	9.0	6.4	5.7	3.9	2.8
Favored (%)	95.5	94.0	91.0	93.6	94.3	96.1	97.2
Rotamer outliers (%)	0	0	0	0	0	0	0
Clash score	2.04	2.39	1.89	0.88	2.50	3.31	1.73
Molprobity score	1.29	1.42	1.47	1.19	1.42	1.40	1.08
FSC model (0/0.143/0.5)	3.3/3.7/4.1	3.3/3.5/3.8	3.5/3.7/4.2	3.0/3.2/3.4	3.3/3.5/3.9	3.3/3.6/4.0	2.9/3.1/3.4
EMRinger score	2.51	2.46	2.04	3.32	2.12	2.44	3.74

Table S2: 12.1F antibody interactions with GP1. Related to Figure 4. Amino acid interactions at the 12.1F epitope-paratope were determined using the online-based Epitope Analyzer platform[12]. Glycan contacts were assessed by finding close contacts (<4 Å) of the GPC glycans with 12.1F Fab using ChimeraX.[7]

GP Residue number	Amino acid	Atom	Fab Residue number	Amino Acid	Heavy or light chain	Distance (Å)	Interaction Type
91	SER	CB-CD2	111.1	PHE	H	3.4	Van-der-Waals
92	HIS	CE1-CD2	111.1	PHE	H	3.9	Van-der-Waals
107	LEU	O-CD2	111.1	PHE	H	3.8	Van-der-Waals
108	THR	C-O	111	GLY	H	3.5	Van-der-Waals
108	THR	CG2-CE2	111.1	PHE	H	3.7	Van-der-Waals
109	ASN	CA-O	111	GLY	H	3.9	Van-der-Waals
109	ASN	N-O	111	GLY	H	2.9	Hydro-Bond
110	THR	CG2-CA	111	GLY	H	3.9	Van-der-Waals
110	THR	CG2-CB	109	SER	H	3.8	Van-der-Waals
111	SER	O-CE2	38	PHE	H	3.9	Van-der-Waals
112	ILE	C-ND2	57	ASN	H	3.7	Van-der-Waals
112	ILE	C-OG	64	SER	H	3.6	Van-der-Waals
112	ILE	CG2-CD1	59	LEU	H	3.5	Hydr-Phbc
112	ILE	O-ND2	57	ASN	H	2.8	Hydro-Bond
112	ILE	O-OG	64	SER	H	2.7	Hydro-Bond
113	ILE	C-OG	64	SER	H	3.4	Van-der-Waals
113	ILE	N-OG	64	SER	H	3.9	Hydro-Bond
114	ASN	CA-OG	64	SER	H	3.6	Van-der-Waals
114	ASN	CB-O	65	THR	H	3.6	Van-der-Waals
114	ASN	N-OG	64	SER	H	2.7	Hydro-Bond
114	ASN	ND2-O	65	THR	H	2.9	Hydro-Bond
156	ASP	CG-CD1	59	LEU	H	3.6	Van-der-Waals
217	TYR	CE2-N	111.2	ALA	H	3.6	Van-der-Waals
217	TYR	OH-C	111.1	PHE	H	4.0	Van-der-Waals
217	TYR	OH-N	111.2	ALA	H	2.9	Hydro-Bond
110	THR	C-OD1	109	ASP	L	4.0	Van-der-Waals
111	SER	CB-NE1	114	TRP	L	4.0	Van-der-Waals
111	SER	CB-OD1	109	ASP	L	3.6	Van-der-Waals
111	SER	N-OD1	109	ASP	L	3.4	Hydro-Bond
111	SER	OG-NE1	114	TRP	L	2.6	Hydro-Bond
111	SER	OG-OD1	109	ASP	L	2.8	Hydro-Bond
111	SER	OG-OD2	109	ASP	L	3.5	Hydro-Bond
112	ILE	C-CZ2	114	TRP	L	3.8	Van-der-Waals
114	ASN	CB-CD2	114	TRP	L	3.8	Van-der-Waals
114	ASN	OD1-NE1	114	TRP	L	3.4	Hydro-Bond
219	TYR	OH-CG	109	ASP	L	3.6	Van-der-Waals
219	TYR	OH-OD1	109	ASP	L	3.9	Hydro-Bond
219	TYR	OH-OD2	109	ASP	L	3.0	Hydro-Bond
N89 glycan, O3-C1 Man		O6-CB	4	LEU	H	3.7	-
N89 glycan, O3-C1 Man		O6-CG	4	LEU	H	3.8	-
N89 glycan, O3-C1 Man		O6-CD1	4	LEU	H	3.9	-
N89 glycan, O3-C1 Man		O6-N	4	LEU	H	3.7	-
N89 glycan, O3-C1 Man		O6-OE2	28	GLU	H	2.8	-
N89 glycan, O3-C1 Man		C6-OE2	28	GLU	H	3.5	-
N89 glycan, O3-C1 Man		O6-CB	28	GLU	H	3.6	-
N89 glycan, O3-C1 Man		OG-CG	28	GLU	H	3.8	-
N89 glycan, O3-C1 Man		C6-CG	28	GLU	H	4.0	-
N89 glycan, O3-C1 Man		O5-OG	29	SER	H	3.2	-
N89 glycan, O3-C1 Man		C6-CB	29	SER	H	3.6	-
N89 glycan, O3-C1 Man		C6-OG	29	SER	H	3.9	-
N89 glycan, O3-C1 Man		O5-CB	29	SER	H	3.7	-
N89 glycan, O4-C1 Man		O3-CZ	37	PHE	H	3.7	-
N89 glycan, O4-C1 GlcNAc		O4-OH	108	TYR	H	2.9	-
N89 glycan, O4-C1 GlcNAc		O3-OH	108	TYR	H	3.1	-
N89 glycan, O4-C1 Man		O2-CE1	108	TYR	H	3.2	-
N89 glycan, O4-C1 Man		O2-CZ	108	TYR	H	3.3	-
N89 glycan, O4-C1 GlcNAc		O4-CZ	108	TYR	H	3.4	-
N89 glycan, O4-C1 Man		O2-CD1	108	TYR	H	3.5	-
N89 glycan, O4-C1 Man		O2-CE2	108	TYR	H	3.7	-
N89 glycan, O4-C1 Man		O2-OH	108	TYR	H	3.8	-
N89 glycan, O4-C1 Man		O2-CD2	108	TYR	H	4.0	-
N89 glycan, O4-C1 GlcNAc		C4-OH	108	TYR	H	3.3	-
N89 glycan, O4-C1 GlcNAc		O4-CE2	108	TYR	H	3.7	-
N89 glycan, O4-C1 GlcNAc		C3-OH	108	TYR	H	3.8	-
N89 glycan, O4-C1 Man		O2-CG2	108	TYR	H	3.9	-
N89 glycan, O4-C1 GlcNAc		O3-CZ	108	TYR	H	3.9	-
N89 glycan, ND2-C1 GlcNAc		O3-OH	110	TYR	H	2.9	-
N89 glycan, ND2-C1 GlcNAc		O3-CZ	110	TYR	H	3.6	-
N89 glycan, ND2-C1 GlcNAc		O7-OH	110	TYR	H	3.2	-
N89 glycan, ND2-C1 GlcNAc		O3-CE1	110	TYR	H	3.5	-
N89 glycan, ND2-C1 GlcNAc		C7-OH	110	TYR	H	3.7	-
N89 glycan, O4-C1 GlcNAc		C6-CE1	110	TYR	H	3.7	-
N89 glycan, ND2-C1 GlcNAc		N2-OH	110	TYR	H	3.9	-
N109 glycan, ND2-C1 GlcNAc		C8-O	111.2	ALA	H	3.0	-
N109 glycan, ND2-C1 GlcNAc		O7-O	111.2	ALA	H	2.9	-
N109 glycan, ND2-C1 GlcNAc		O7-O	111.2	ALA	H	3.2	-
N109 glycan, ND2-C1 GlcNAc		C8-C	111.2	ALA	H	3.9	-
N109 glycan, ND2-C1 GlcNAc		O3-CE3	112	TRP	H	3.9	-
N109 glycan, O4-C1 GlcNAc		C5-CE2	112	TRP	H	3.9	-
N109 glycan, O4-C1 GlcNAc		C6-NE1	112	TRP	H	4.0	-
N109 glycan, ND2-C1 GlcNAc		O7-ND2	112.1	ASN	H	3.9	-
N109 glycan, ND2-C1 GlcNAc		O7-CE3	112.3	TRP	H	3.8	-
N109 glycan, O4-C1 GlcNAc		C5-CZ2	112.3	TRP	H	3.9	-
N89 glycan, O3-C1 Man		O4-CG2	116	ASP	H	3.9	-
N89 glycan, O3-C1 Man		O3-CB	116	ASP	H	3.6	-
N89 glycan, O3-C1 Man		O4-CB	116	ASP	H	3.8	-
N89 glycan, O3-C1 Man		O4-CG2	117	VAL	H	3.2	-
N89 glycan, O3-C1 Man		O6-CG2	117	VAL	H	3.3	-
N109 glycan, ND2-C1 GlcNAc		O6-OG	36	SER	L	3.9	-
N109 glycan, ND2-C1 GlcNAc		C6-OG	36	SER	L	4.0	-
N89 glycan, O3-C1 Man		O2-OG1	69	THR	L	2.5	-
N89 glycan, O3-C1 Man		O2-CG2	69	THR	L	3.5	-
N89 glycan, O3-C1 Man		C2-CG2	69	THR	L	3.8	-
N89 glycan, O6-C1 Man		O2-CG2	69	THR	L	3.3	-
N89 glycan, O3-C1 Man		O2-CB	69	THR	L	3.5	-
N89 glycan, O3-C1 Man		C2-OG1	69	THR	L	3.6	-
N167 glycan, ND2-C1 GlcNAc		C8-OD2	109	ASP	L	3.7	-

Table S3: 19.7E antibody interactions with GP1. Related to Figure 4. Amino acid interactions at the 19.7E epitope-paratope region were determined using the online-based Epitope Analyzer platform.[12] Glycan contacts were assessed by finding close contacts (<4 Å) of the GPC glycans with 19.7E Fab using ChimeraX[7].

GP Residue number	Amino acid	Atom	Fab Residue number	Amino Acid	Heavy or light chain	Distance (Å)	Interaction Type
92	HIS	CE1-OE2	1	GLU	H	3.5	Van-der-Waals
104	GLU	CD-OE1	1	GLU	H	3.8	Van-der-Waals
109	ASN	C-CB	114	SER	H	3.7	Van-der-Waals
109	ASN	C-OD1	112	ASP	H	3.8	Van-der-Waals
109	ASN	CB-CB	113	TRP	H	4.0	Van-der-Waals
109	ASN	ND2-O	113	TRP	H	3.8	Hydro-Bond
109	ASN	O-N	113	TRP	H	3.4	Hydro-Bond
109	ASN	O-N	114	SER	H	2.9	Hydro-Bond
110	THR	C-OD2	112	ASP	H	3.7	Van-der-Waals
110	THR	CG2-CB	114	SER	H	3.7	Van-der-Waals
111	SER	C-OH	37	TYR	H	3.5	Van-der-Waals
111	SER	CA-OD2	112	ASP	H	3.6	Van-der-Waals
111	SER	N-OD1	112	ASP	H	3.8	Hydro-Bond
111	SER	N-OD2	112	ASP	H	2.8	Hydro-Bond
111	SER	O-CZ	107	ARG	H	3.7	Van-der-Waals
111	SER	O-NH1	107	ARG	H	3.5	Hydro-Bond
111	SER	O-NH2	107	ARG	H	3.1	Hydro-Bond
111	SER	O-OH	37	TYR	H	3.7	Hydro-Bond
111	SER	OG-NH1	107	ARG	H	3.8	Hydro-Bond
111	SER	OG-OD2	112	ASP	H	2.8	Hydro-Bond
111	SER	OG-OH	37	TYR	H	2.8	Hydro-Bond
112	ILE	C-CE1	37	TYR	H	3.7	Van-der-Waals
112	ILE	CA-CB	28	PHE	H	3.9	Hydr-Phbc
112	ILE	CB-CB	28	PHE	H	3.9	Hydr-Phbc
112	ILE	CG2-CG2	2	VAL	H	3.9	Hydr-Phbc
112	ILE	CG2-O	27	GLY	H	3.9	Van-der-Waals
112	ILE	N-OH	37	TYR	H	3.4	Hydro-Bond
112	ILE	O-CA	29	SER	H	3.7	Van-der-Waals
112	ILE	O-N	29	SER	H	2.9	Hydro-Bond
112	ILE	O-OG	29	SER	H	3.8	Hydro-Bond
112	ILE	O-OH	37	TYR	H	3.4	Hydro-Bond
113	ILE	C-OH	37	TYR	H	3.4	Van-der-Waals
113	ILE	CG1-OG	29	SER	H	3.5	Van-der-Waals
113	ILE	N-OH	37	TYR	H	3.0	Hydro-Bond
113	ILE	O-OH	37	TYR	H	3.8	Hydro-Bond
114	ASN	CB-CE2	37	TYR	H	3.7	Van-der-Waals
114	ASN	CG-C	36	SER	H	4.0	Van-der-Waals
114	ASN	N-OH	37	TYR	H	3.4	Hydro-Bond
114	ASN	ND2-O	36	SER	H	3.0	Hydro-Bond
114	ASN	OD1-N	37	TYR	H	3.9	Hydro-Bond
114	ASN	OD1-OH	37	TYR	H	4.0	Hydro-Bond
115	HIS	CB-OG	36	SER	H	3.8	Van-der-Waals
115	HIS	ND1-OG	36	SER	H	3.7	Hydro-Bond
156	ASP	CB-OG	29	SER	H	3.4	Van-der-Waals
156	ASP	OD2-OG	29	SER	H	2.8	Hydro-Bond
169	SER	CB-CZ2	113	TRP	H	4.0	Van-der-Waals
172	TYR	CE1-CH2	113	TRP	H	4.0	Hydr-Phbc
216	SER	O-CZ3	113	TRP	H	4.0	Van-der-Waals
217	TYR	C-CE3	113	TRP	H	4.0	Van-der-Waals
218	GLN	CA-CE3	113	TRP	H	3.8	Van-der-Waals
218	GLN	NE2-O	111	TYR	H	3.1	Hydro-Bond
218	GLN	OE1-C	112	ASP	H	3.7	Van-der-Waals
218	GLN	OE1-N	113	TRP	H	2.8	Hydro-Bond
223	GLN	NE2-OE1	1	GLU	H	3.3	Hydro-Bond
223	GLN	OE1-O	27	GLY	H	3.5	Van-der-Waals
N89 glycan, O4-C1 GlcNAc		O7-CD	1	GLU	H	4.0	-
N89 glycan, O4-C1 GlcNAc		O7-CG	1	GLU	H	3.2	-
N167, O4-C1 GlcNac		O6-CE1	111	TYR	H	3.3	-
N167, O4-C1 GlcNac		O6-CD1	111	TYR	H	3.5	-
N167, O4-C1 GlcNac		O7-CD2	111	TYR	H	3.6	-
N167 glycan, O4-C1 Man		C1-OH	111	TYR	H	3.7	-
N167 glycan, O4-C1 Man		C3-OH	111	TYR	H	3.7	-
N167, O4-C1 GlcNac		C2-CD2	111	TYR	H	3.9	-
N109 glycan, ND2-C1 GlcNAc		C1-CE3	113	TRP	H	3.7	-
N109 glycan, ND2-C1 GlcNAc		O6-CD2	113	TRP	H	3.8	-
N109 glycan, ND2-C1 GlcNAc		O6-CE2	113	TRP	H	3.8	-
N109 glycan, ND2-C1 GlcNAc		O6-CD1	113	TRP	H	3.9	-
N109 glycan, ND2-C1 GlcNAc		O5-CE3	113	TRP	H	3.9	-
N109 glycan, ND2-C1 GlcNAc		O6-NE1	113	TRP	H	3.9	-
N109 glycan, ND2-C1 GlcNAc		O5-CB	113	TRP	H	3.8	-
N109 glycan, ND2-C1 GlcNAc		O6-CG	113	TRP	H	3.8	-
N109 glycan, ND2-C1 GlcNAc		C1-CB	113	TRP	H	4.0	-
N89 glycan, O6-C1 Man		O4-CG	118	TRP	H	3.6	-
N89 glycan, O6-C1 Man		O4-CD1	118	TRP	H	3.9	-
N89 glycan, O6-C1 Man		O4-CB	118	TRP	H	3.6	-
N89 glycan, O6-C1 Man		C6-CB	49	ALA	L	3.7	-
N89 glycan, O6-C1 Man		O6-CB	49	ALA	L	3.8	-
N89 glycan, O6-C1 Man		O2-CG	51	LYS	L	3.8	-
N167 glycan, O3-C1 Man		C6-OD1	108	ASN	L	3.1	-
N167 glycan, O3-C1 Man		O4-C	108	ASN	L	3.5	-
N167 glycan, O3-C1 Man		C4-O	108	ASN	L	3.8	-
N167 glycan, O3-C1 Man		O6-OD1	108	ASN	L	3.9	-
N167 glycan, O3-C1 Man		C6-CG	108	ASN	L	4.0	-

Table S4: S370.7 antibody interactions with GPC. Related to Figure 6. Amino acid interactions at the S370.7 epitope-paratope region were determined using the online-based Epitope Analyzer platform.[12] Glycan contacts were assessed by finding close contacts (<4 Å) of the GPC glycans with S370.7 Fab using ChimeraX.[7]

GP Residue number	Amino acid	Atom	Fab Residue number	Amino Acid	Heavy or light chain	Distance (Å)	Interaction Type
268	ASP	CB-CG	112.1	PRO	H	4.0	Van-der-Waals
268	ASP	OD2-CB	111.1	SER	H	3.9	Van-der-Waals
325	ARG	NH1-CB	112.4	VAL	H	3.7	Van-der-Waals
325	ARG	NH1-O	112.4	VAL	H	3.7	Hydro-Bond
325	ARG	NH1-OG	64	SER	H	3.8	Hydro-Bond
325	ARG	NH2-O	112.4	VAL	H	3.8	Hydro-Bond
360	CYS	O-CG1	112.4	VAL	H	3.6	Van-der-Waals
362	PRO	CD-CD2	112.5	TYR	H	3.9	Hydr-Phbc
362	PRO	CG-CD2	112.5	TYR	H	3.4	Hydr-Phbc
362	PRO	CG-CE2	112.5	TYR	H	3.8	Hydr-Phbc
387	LEU	CD1-CG1	111.2	VAL	H	3.7	Hydr-Phbc
387	LEU	CD1-CG2	111.2	VAL	H	4.0	Hydr-Phbc
389	SER	OG-CE2	112.5	TYR	H	3.9	Van-der-Waals
394	LEU	CD2-CE1	112.5	TYR	H	3.9	Hydr-Phbc
394	LEU	CD2-CE2	112.5	TYR	H	3.9	Hydr-Phbc
394	LEU	CD2-CZ	112.5	TYR	H	3.6	Hydr-Phbc
397	THR	O-CG	111	ARG	H	3.8	Van-der-Waals
398	HIS	C-C	111	ARG	H	3.8	Van-der-Waals
398	HIS	CD2-CD2	58	HIS	H	3.9	Van-der-Waals
398	HIS	CG-CE1	112.5	TYR	H	3.9	Van-der-Waals
398	HIS	O-C	111.1	SER	H	3.4	Van-der-Waals
398	HIS	O-N	111.1	SER	H	3.5	Hydro-Bond
399	PHE	C-O	111	ARG	H	3.1	Van-der-Waals
399	PHE	CB-CG2	111.2	VAL	H	3.8	Hydr-Phbc
399	PHE	N-O	111	ARG	H	3.5	Hydro-Bond
400	SER	C-O	111	ARG	H	3.8	Van-der-Waals
400	SER	N-O	111	ARG	H	3.0	Hydro-Bond
401	ASP	CA-O	111	ARG	H	3.6	Van-der-Waals
401	ASP	N-O	111	ARG	H	2.9	Hydro-Bond
401	ASP	OD2-N	111	ARG	H	3.7	Hydro-Bond
402	ASP	CG-CB	111.1	SER	H	4.0	Van-der-Waals
402	ASP	CG-N	111.2	VAL	H	3.9	Van-der-Waals
402	ASP	OD2-N	111.2	VAL	H	2.9	Hydro-Bond
269	SER	OG-CE	36	LYS	L	3.3	Van-der-Waals
269	SER	OG-NZ	36	LYS	L	3.0	Hydro-Bond
270	GLU	O-CG	36	LYS	L	3.4	Van-der-Waals
272	LYS	CA-OH	38	TYR	L	3.9	Van-der-Waals
272	LYS	CB-O	29	PRO	L	4.0	Van-der-Waals
272	LYS	CD-C	37	GLN	L	3.8	Van-der-Waals
272	LYS	CD-O	28	LEU	L	4.0	Van-der-Waals
272	LYS	CD-OD2	57	ASP	L	3.4	Van-der-Waals
272	LYS	CG-C	36	LYS	L	3.9	Van-der-Waals
272	LYS	N-O	29	PRO	L	3.5	Hydro-Bond
272	LYS	N-O	36	LYS	L	3.9	Hydro-Bond
272	LYS	NZ-O	28	LEU	L	2.6	Hydro-Bond
272	LYS	NZ-O	37	GLN	L	3.1	Hydro-Bond
272	LYS	NZ-OD1	57	ASP	L	3.6	Salt-Bridge
272	LYS	NZ-OD2	57	ASP	L	2.8	Salt-Bridge
272	LYS	O-OH	38	TYR	L	3.5	Hydro-Bond
279	CYS	CB-CD	36	LYS	L	3.9	Van-der-Waals
281	THR	CB-OD1	110	ASP	L	3.5	Van-der-Waals
281	THR	CG2-C	109	SER	L	3.9	Van-der-Waals
281	THR	CG2-NZ	36	LYS	L	3.8	Van-der-Waals
281	THR	OG1-OD1	110	ASP	L	2.9	Hydro-Bond
283	TRP	CH2-CB	109	SER	L	3.9	Van-der-Waals
283	TRP	CZ3-CG	110	ASP	L	3.9	Van-der-Waals
290	LEU	CD2-CB	29	PRO	L	4.0	Hydr-Phbc
290	LEU	CD2-CB	36	LYS	L	3.9	Van-der-Waals
290	LEU	CD2-O	26	ASP	L	3.9	Van-der-Waals
320	LYS	CE-O	110	ASP	L	4.0	Van-der-Waals
320	LYS	NZ-O	110	ASP	L	3.8	Hydro-Bond
324	GLN	NE2-OG1	114	THR	L	3.1	Hydro-Bond
N390 glycan, ND2-C1 GlcNAc		C8-O	59	SER	H	3.2	-
N390 glycan, ND2-C1 GlcNAc		C8-C	59	SER	H	3.8	-
N390 glycan, ND2-C1 GlcNAc		C8-CA	63	GLY	H	4.1	-
N79 glycan, O3-C1 Man		O4-CE2	115	TYR	L	3.2	-
N79 glycan, O3-C1 Man		O4-CD2	115	TYR	L	3.4	-
N79 glycan, O3-C1 Man		C5-N	2	TYR	L	3.9	-
N79 glycan, O3-C1 Man		O4-N	2	TYR	L	3.0	-

Table S5: 37.2D antibody interactions with GPC. Related to Figure 6. Amino acid interactions at the 37.2D epitope-paratope region (PDB 7UOT)[10] were determined using the online-based Epitope Analyzer platform.[12] Glycan contacts were assessed by finding close contacts (<4 Å) of the GPC glycans with 37.2D Fab using ChimeraX.[7]

GP Residue number	Amino acid	Atom	Fab Residue number	Amino Acid	Heavy or light chain	Distance (Å)	Interaction Type
264	TRP	CH2-CH2	112.3	TRP	H	3.8	Hydr-Phbc
264	TRP	CH2-CZ2	112.3	TRP	H	4.0	Hydr-Phbc
264	TRP	CZ2-CH2	112.3	TRP	H	3.7	Hydr-Phbc
264	TRP	CZ2-CZ2	112.3	TRP	H	3.5	Hydr-Phbc
265	THR	O-NE1	112.3	TRP	H	3.5	Hydro-Bond
267	SER	CA-CD1	112.3	TRP	H	3.4	Van-der-Waals
321	GLN	CG-CH2	112.3	TRP	H	3.8	Van-der-Waals
325	ARG	NE-CZ3	112.3	TRP	H	3.8	Van-der-Waals
62	TYR	CZ-CZ	59	PHE	H	3.8	Hydr-Phbc
352	LYS	NZ-O	111.3	SER	H	3.6	Hydro-Bond
356	ARG	CZ-O	111.3	SER	H	3.2	Van-der-Waals
356	ARG	CZ-O	112.4	GLY	H	3.4	Van-der-Waals
356	ARG	NE-O	111.3	SER	H	3.7	Hydro-Bond
356	ARG	NH1-O	111.3	SER	H	3.7	Hydro-Bond
356	ARG	NH1-O	112.4	GLY	H	3.4	Hydro-Bond
356	ARG	NH2-O	111.2	SER	H	2.9	Hydro-Bond
356	ARG	NH2-O	111.3	SER	H	3.0	Hydro-Bond
356	ARG	NH2-O	112.4	GLY	H	2.5	Hydro-Bond
361	ILE	CD1-CE3	112.3	TRP	H	3.5	Hydr-Phbc
361	ILE	CD1-CZ3	112.3	TRP	H	3.4	Hydr-Phbc
362	PRO	CD-CD1	111.1	TYR	H	3.7	Hydr-Phbc
362	PRO	CD-CE1	111.1	TYR	H	3.9	Hydr-Phbc
362	PRO	CG-CD1	111.1	TYR	H	3.5	Hydr-Phbc
362	PRO	CG-CE1	111.1	TYR	H	3.8	Hydr-Phbc
362	PRO	CG-CG	111.1	TYR	H	3.8	Hydr-Phbc
384	LYS	NZ-OG	111.3	SER	H	3.6	Hydro-Bond
387	LEU	CD1-C	111.2	SER	H	3.8	Van-der-Waals
394	LEU	CD1-CB	111.1	TYR	H	3.8	Hydr-Phbc
394	LEU	CD1-CD2	111.1	TYR	H	3.6	Hydr-Phbc
394	LEU	CD1-CG	111.1	TYR	H	3.9	Hydr-Phbc
394	LEU	CD2-CD2	111.1	TYR	H	3.9	Hydr-Phbc
395	ASN	ND2-CD1	64	TYR	H	3.5	Van-der-Waals
396	GLU	OE1-ND2	62	ASN	H	3.9	Hydro-Bond
397	THR	CB-ND2	62	ASN	H	3.7	Van-der-Waals
397	THR	CB-OG	52	SER	H	3.7	Van-der-Waals
397	THR	CG2-CB	64	TYR	H	4.0	Van-der-Waals
397	THR	O-CB	109	PRO	H	3.4	Van-der-Waals
397	THR	O-NE2	111	GLN	H	3.4	Hydro-Bond
397	THR	OG1-OG	57	SER	H	2.7	Hydro-Bond
398	HIS	C-O	110	ASP	H	3.7	Van-der-Waals
398	HIS	C-OE1	111	GLN	H	3.9	Van-der-Waals
398	HIS	CE1-CH2	55	TRP	H	4.0	Van-der-Waals
398	HIS	ND1-O	110	ASP	H	2.8	Hydro-Bond
398	HIS	NE2-CE2	64	TYR	H	3.8	Van-der-Waals
398	HIS	NE2-NH1	66	ARG	H	3.5	Van-der-Waals
398	HIS	O-CA	111.1	TYR	H	4.0	Van-der-Waals
398	HIS	O-N	111.1	TYR	H	3.0	Hydro-Bond
399	PHE	C-OE1	111	GLN	H	3.7	Van-der-Waals
399	PHE	CA-O	111.1	TYR	H	3.9	Van-der-Waals
399	PHE	N-OE1	111	GLN	H	4.0	Hydro-Bond
400	SER	N-OE1	111	GLN	H	3.4	Hydro-Bond
401	ASP	CA-OE1	111	GLN	H	3.7	Van-der-Waals
401	ASP	CG-OH	108	TYR	H	3.8	Van-der-Waals
401	ASP	N-OE1	111	GLN	H	3.0	Hydro-Bond
401	ASP	OD1-OH	108	TYR	H	3.1	Hydro-Bond
401	ASP	OD2-CZ	112.1	ARG	H	4.0	Van-der-Waals
401	ASP	OD2-NH1	112.1	ARG	H	3.9	Salt-Bridge
401	ASP	OD2-NH2	112.1	ARG	H	3.0	Salt-Bridge
401	ASP	OD2-OH	108	TYR	H	3.8	Hydro-Bond
404	GLU	CD-NZ	36	LYS	H	3.2	Van-der-Waals
404	GLU	OE1-NZ	36	LYS	H	3.1	Salt-Bridge
404	GLU	OE2-NZ	36	LYS	H	2.6	Salt-Bridge
324	GLN	C-CG2	36	ILE	L	3.9	Van-der-Waals
324	GLN	C-ND2	28	ASN	L	4.0	Van-der-Waals
324	GLN	O-ND2	28	ASN	L	2.8	Hydro-Bond
325	ARG	CZ-CD1	36	ILE	L	3.5	Van-der-Waals
325	ARG	O-ND2	28	ASN	L	3.9	Hydro-Bond
N390 glycan, O4-C1 GlcNac		O6-NE2	72	GLN	H	3.8	-
N395 glycan, ND2-C1 GlcNac		C5-OD1	62	ASN	H	3.8	-
N395 glycan, ND2-C1 GlcNac		C6-OD1	62	ASN	H	4	-
N395 glycan, ND2-C1 GlcNac		C5-CG	62	ASN	H	3.9	-
N395 glycan, ND2-C1 GlcNac		C6-O	62	ASN	H	3.6	-
N395 glycan, ND2-C1 GlcNac		O5-OD1	62	ASN	H	3.6	-
N395 glycan, ND2-C1 GlcNac		C6-C	63	GLY	H	3.8	-
N395 glycan, ND2-C1 GlcNac		C6-N	64	TYR	H	3.7	-
N395 glycan, ND2-C1 GlcNac		O5-CB	64	TYR	H	3.8	-
N395 glycan, O4-C1 GlcNac		C8-O	62	ASN	H	3.3	-
N395 glycan, C6-O6 Fuc		C1-O	63	GLY	H	3.5	-
N395 glycan, C6-O6 Fuc		O5-OD1	63	GLY	H	3.6	-
N395 glycan, C6-O6 Fuc		C6-CE1	64	TYR	H	4	-
N395 glycan, C6-O6 Fuc		O5-CA	64	TYR	H	3.8	-
N395 glycan, C6-O6 Fuc		C6-CD1	64	TYR	H	3.9	-
N395 glycan, C6-O6 Fuc		C6-OG1	65	THR	H	3.9	-
N395 glycan, C6-O6 Fuc		O5-N	65	THR	H	4	-

References

1. Notredame, C., Higgins, D.G., and Heringa, J. (2000). T-Coffee: A novel method for fast and accurate multiple sequence alignment. J. Mol. Biol. *302*, 205–217. 10.1006/jmbi.2000.4042.

2. Cohen-Dvashi, H., Cohen, N., Israeli, H., and Diskin, R. (2015). Molecular Mechanism for LAMP1 Recognition by Lassa Virus. J. Virol. *89*, 7584–7592. 10.1128/JVI.00651-15.

3. Israeli, H., Cohen-Dvashi, H., Shulman, A., Shimon, A., and Diskin, R. (2017). Mapping of the Lassa virus LAMP1 binding site reveals unique determinants not shared by other old world arenaviruses. PLOS Pathog. *13*, e1006337.

4. Hastie, K.M., Zandonatti, M.A., Kleinfelter, L.M., Heinrich, M.L., Rowland, M.M., Chandran, K., Branco, L.M., Robinson, J.E., Garry, R.F., and Saphire, E.O. (2017). Structural basis for antibody-mediated neutralization of Lassa virus. Science *356*, 923–928. 10.1126/science.aam7260.

5. Robert, X., and Gouet, P. (2014). Deciphering key features in protein structures with the new ENDscript server. Nucleic Acids Res. *42*, W320-4. 10.1093/nar/gku316.

6. Punjani, A., Rubinstein, J.L., Fleet, D.J., and Brubaker, M.A. (2017). cryoSPARC: algorithms for rapid unsupervised cryo-EM structure determination. Nat. Methods *14*, 290–296. 10.1038/nmeth.4169.

7. Pettersen, E.F., Goddard, T.D., Huang, C.C., Meng, E.C., Couch, G.S., Croll, T.I., Morris, J.H., and Ferrin, T.E. (2021). UCSF ChimeraX: Structure visualization for researchers, educators, and developers. Protein Sci. *30*, 70–82. 10.1002/pro.3943.

8. Katz, M., Weinstein, J., Eilon-Ashkenazy, M., Gehring, K., Cohen-Dvashi, H., Elad, N., Fleishman, S.J., and Diskin, R. (2022). Structure and receptor recognition by the Lassa virus spike complex. Nature. 10.1038/s41586-022-04429-2.

9. Enriquez, A.S., Buck, T.K., Li, H., Norris, M.J., Moon-Walker, A., Zandonatti, M.A., Harkins, S.S., Robinson, J.E., Branco, L.M., Garry, R.F., et al. (2022). Delineating the mechanism of anti-Lassa virus GPC-A neutralizing antibodies. Cell Rep. *39*, 110841. https://doi.org/10.1016/j.celrep.2022.110841.

10. Li, H., Buck, T., Zandonatti, M., Yin, J., Moon-Walker, A., Fang, J., Koval, A., Heinrich, M.L., Rowland, M.M., Diaz Avalos, R., et al. (2022). A cocktail of protective antibodies subverts the dense glycan shield of Lassa virus. Sci. Transl. Med. *14*, eabq0991. 10.1126/scitranslmed.abq0991.

11. Hastie, K.M., Cross, R.W., Harkins, S.S., Zandonatti, M.A., Koval, A.P., Heinrich, M.L., Rowland, M.M., Robinson, J.E., Geisbert, T.W., Garry, R.F., et al. (2019). Convergent Structures Illuminate Features for Germline Antibody Binding and Pan-Lassa Virus Neutralization. Cell *178*, 1004-1015.e14. 10.1016/j.cell.2019.07.020.

12. Montiel-Garcia, D., Rojas-Labra, O., Santoyo-Rivera, N., and Reddy, V.S. (2022). Epitope-Analyzer: A structure-based webtool to analyze broadly neutralizing epitopes. J. Struct. Biol. *214*, 107839. 10.1016/j.jsb.2022.107839.
